# Modeling the heterogeneity of sodium and calcium homeostasis between cortical and hippocampal astrocytes and its impact on bioenergetics

**DOI:** 10.3389/fncel.2023.1035553

**Published:** 2023-01-30

**Authors:** Pawan Thapaliya, Nils Pape, Christine R. Rose, Ghanim Ullah

**Affiliations:** ^1^Department of Physics, University of South Florida, Tampa, FL, United States; ^2^Faculty of Mathematics and Natural Sciences, Institute of Neurobiology, Heinrich Heine University Düsseldorf, Düsseldorf, Germany

**Keywords:** bioenergetics, ATP, astrocytic Na^+^ signaling, astrocytic Ca^2+^ signaling, heterogeneity in astrocytic Na^+^

## Abstract

Emerging evidence indicates that neuronal activity-evoked changes in sodium concentration in astrocytes Na_*a*_ represent a special form of excitability, which is tightly linked to all other major ions in the astrocyte and extracellular space, as well as to bioenergetics, neurotransmitter uptake, and neurovascular coupling. Recently, one of us reported that Na_*a*_ transients in the neocortex have a significantly higher amplitude than those in the hippocampus. Based on the extensive data from that study, here we develop a detailed biophysical model to further understand the origin of this heterogeneity and how it affects bioenergetics in the astrocytes. In addition to closely fitting the observed experimental Na_*a*_ changes under different conditions, our model shows that the heterogeneity in Na_*a*_ signaling leads to substantial differences in the dynamics of astrocytic Ca^2+^ signals in the two brain regions, and leaves cortical astrocytes more susceptible to Na^+^ and Ca^2+^ overload under metabolic stress. The model also predicts that activity-evoked Na_*a*_ transients result in significantly larger ATP consumption in cortical astrocytes than in the hippocampus. The difference in ATP consumption is mainly due to the different expression levels of NMDA receptors in the two regions. We confirm predictions from our model experimentally by fluorescence-based measurement of glutamate-induced changes in ATP levels in neocortical and hippocampal astrocytes in the absence and presence of the NMDA receptor's antagonist (2R)-amino-5-phosphonovaleric acid.

## Introduction

Since the discovery of glutamate-induced changes in astrocytic calcium concentration (Ca_*a*_), Ca^2+^ homeostasis in astrocytes has been the subject of intense research in many labs (Cornell-Bell et al., 1990). The role of astrocytic Ca^2+^ signaling in diverse mechanisms which are crucial to normal brain function, such as gliotransmission, tissue volume, bioenergetics, and neurovascular coupling has been extensively studied (for example, see Bazargani and Attwell, 2016; Verkhratsky and Nedergaard, 2018 for review). Thus, in the past, the role of astrocytic ionic signaling was synonymous with the role of astrocytic Ca^2+^ signaling in health and disease (Petzold and Murthy, 2011; Volterra et al., 2014; Rusakov, 2015; Guerra-Gomes et al., 2018; Verkhratsky et al., 2019). However, emerging evidence shows that neuronal activity also evokes transient increases in astrocytic sodium concentration (Na_*a*_) that can regulate several astrocytic functions such as neurotransmitter uptake, K^+^ buffering, pH, and the spatiotemporal dynamics of Ca_*a*_ itself (Rose and Ransom, 1996; Kelly et al., 2009; Langer and Rose, 2009; Attwell et al., 2010; Kirischuk et al., 2012; Parpura and Verkhratsky, 2012; Rose and Karus, 2013; Chatton et al., 2016; Rose and Verkhratsky, 2016; Rose et al., 2018a,b, 2020; Gerkau et al., 2019; Ziemens et al., 2019; Felix et al., 2020; Verkhratsky et al., 2020). Therefore, gaining a deeper insight into this relatively understudied aspect of astrocytic signaling is crucial to understanding the role of astrocytes in health and disease.

Another largely ignored aspect of astrocytic function is the potential differences in their ion homeostasis from one brain region to another. There is strong evidence that the number of glutamate and GABA transporters in astrocytes significantly differs among different brain regions, leading to differences in neurotransmitter uptake, and consequently the overall excitability of the tissue (Lehre and Danbolt, 1998; Danbolt, 2001; Zhou and Danbolt, 2013; Scimemi, 2014; Ortega and Schousboe, 2017; Rose et al., 2018a; Schousboe, 2019) (see also Rusnakova et al., 2013). Furthermore, Lalo et al. (2006) showed that neocortical astrocytes express functional NMDA (and AMPA) receptors that can be activated by both NMDA and glutamate. This is in contrast to the hippocampal CA1 area, in which astrocytes do not show prominent expression of these receptors (Matthias et al., 2003). The differences in the expression levels of NMDA and AMPA receptors were shown to cause a heterogeneity in astrocytic Na^+^ and Ca^2+^ homeostasis between the hippocampus and neocortex (Ziemens et al., 2019). Thus, understanding the molecular mechanisms underlying the differences in Na^+^ and Ca^2+^ homeostasis in different brain regions is crucial to understanding how the crosstalk between astrocytes and neurons differs from one region to another.

Accordingly, the main goal of this study is to develop a detailed biophysical model replicating key observations about Na^+^ and Ca^2+^ homeostasis in cortical and hippocampal astrocytes. Specifically, we aim to provide deeper insight into the role of NMDA and AMPA receptors in the observed heterogeneity in Na^+^ and Ca^2+^ homeostasis in astrocytes in the two brain regions and the downstream effects of this heterogeneity. In the later part of the paper, we focus on the effect of heterogenous expression levels of NMDA receptors on Na^+^ and Ca^2+^ dynamics and ATP consumption in the cortical and hippocampal astrocytes when stimulated by a high concentration of glutamate or NMDA with the activity of Na^+^/K^+^-ATPase suppressed as would be the case in numerous pathologies such as ischemic stroke, migraine, and traumatic brain injury (Hansen and Zeuthen, 1981; Dreier, 2011; Ayata and Lauritzen, 2015; Enger et al., 2015; Hu and Song, 2017; Murata et al., 2020; Andrew et al., 2022).

## Materials and methods

**Figure 1:**
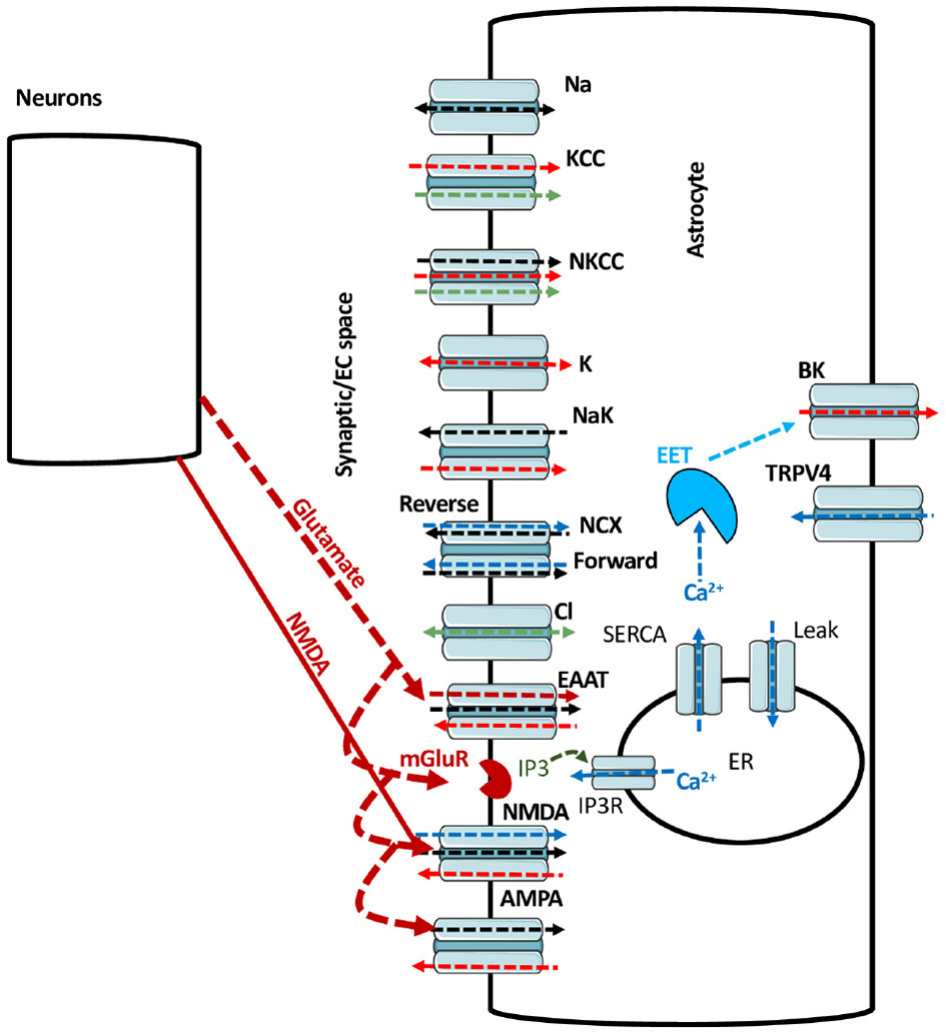
A schematic of the main pathways incorporated in model. The black, red, blue, green, and maroon arrows represent Na^+^, K^+^, Ca^2+^, Cl^-^, and glutamate fluxes respectively. The arrow heads represents the direction of the flux.

A schematic of the astrocytic model is shown in Figure 1. Glutamate added to the bath solution in *in vitro* experiments or applied to the cell in the model is bound by glutamate transporters, and activates metabotropic glutamate receptors (mGluR) to generate inositol 1,4,5-trisphosphate (IP_3_). IP_3_ activates IP_3_ receptors (IP_3_Rs) on the endoplasmic reticulum (ER) to release Ca^2+^. ER also releases Ca^2+^ to the cytoplasm through leak channels and buffers Ca^2+^ through sarco/ER Ca^2+^-ATPase (SERCA). With the import of each glutamate molecule, a glutamate transporter (excitatory amino acid transporter; EAAT) exports one K^+^ and imports three Na^+^ and one H^+^ into the astrocyte. Glutamate also activates NMDA and AMPA receptors. NMDA receptors allow Na^+^ and Ca^2+^ to enter while K^+^ leaves the cell. AMPA receptors imports Na^+^ into the cell while exporting K^+^. In the forward mode, Na^+^/Ca^2+^ exchanger (NCX) allows three Na^+^ to enter and one Ca^2+^ to leave the astrocyte. In reverse mode, NCX exports three Na^+^ to the extracellular space (ECS) and imports one Ca^2+^ into the astrocyte (Rose et al., 2020). With the consumption of one ATP, Na^+^/K^+^-ATPase exports three Na^+^ from the astrocyte in exchange for two K^+^. The rise in Ca_*a*_ causes the production of epoxyeicosatrienoic acid (EET), which together with Ca^2+^ opens BK channels to release K^+^ to the ECS. The model also incorporates the exchange of Na^+^, K^+^, Ca^2+^, and Cl^-^ between the ECS and astrocyte through leak Na^+^ channels, K^+^/Cl^-^ co-transporters (KCC), Na^+^/K^+^/Cl^-^ co-transporters (NKCC), K^+^ leak channels, Cl^-^ leak channels, TRPV4, and BK channels.

The model builds on the work in Kenny et al. (2018a,b), and is updated by including NCX, glutamate transporters, NMDA and AMPA receptors. Equations for NCX and glutamate transporters are taken from Oschmann (2018). Equations for astrocytic NMDA and AMPA receptors are developed in this work as described in the Results section. Unless mentioned otherwise, 1 mM glutamate for 100 ms and 0.5 mM NMDA for 50 ms are applied to investigate the glutamate- and NMDA-mediated fluxes in astrocytes. After 100 ms, glutamate decays following the function $Glu_{max}exp\left(-\frac{t-t_{glurlx}}{\tau_{glu}}\right)$, where Glu_*max*_ (mM), t_*glurlx*_, and τ_*glu*_ (s) is the maximum glutamate concentration, starting time for glutamate decay and decay time constant, respectively. Similarly, after 50 ms, NMDA decays according to the function $NMDA_{max}exp\left(-\frac{t-t_{NMDArlx}}{\tau_{NMDA}}\right)$, where NMDA_*max*_ (mM), t_*NMDArlx*_ and τ_*NMDA*_ (s) is the maximum NMDA concentration, starting time for NMDA decay and decay time constant, respectively.

We investigate the role of the reverse mode of NCX in Na^+^ and Ca^2+^ dynamics by considering the effect of 50 mM NMDA in the iontophoresis pipette as was used in Ziemens et al. (2019). It should be noted that in the case of iontophoresis, the agonist concentration applied to the tissue does not reach the concentration in the pipette because the agonist is not directly applied into the tissue. Instead, a current is used to drive the agonist out of the pipette. Thus, the resulting concentration of the agonist in the tissue is unknown but less than that in the pipette. To take this effect into consideration, in this set of simulations, we expose the astrocyte to 4 mM NMDA concentration for 50 ms.

To match the resting Na^+^ concentration observed in Ziemens et al. (2019), we assumed the strength of Na^+^/K^+^-ATPase in the cortex to be slightly lower than the hippocampus. This difference could be due to the heterogeneity in the expression of Na^+^/K^+^-ATPase across different brain regions (Blanco, 2005; Verkhratsky and Nedergaard, 2018; Murata et al., 2020). It should be noted that in the presence of glutamate or NMDA the Na^+^/K^+^-ATPase activity in the cortex is significantly higher than the hippocampus as we will see in the Results section. Furthermore, we take the agonist concentration in the model to be zero in the resting state. Thus, the NMDA and AMPA receptors are closed in both brain regions. While one would expect a non-zero ambient agonist concentration in the tissue, this assumption is valid here as we are mostly interested in the relative changes in the intracellular Na^+^ and Ca^2+^ concentrations as the ligand concentration is raised by a fixed amount above the resting value. An alternative approach would be to assume the same Na^+^/K^+^ pump strengths with non-zero ligand concentration that differs between the two regions in the resting state. However, this does not change the main conclusions of the study (not shown). Furthermore, there is a clear evidence in the literature for the heterogeneous expression of Na^+^/K^+^-ATPase across different brain regions. Thus, we hypothesize the Na^+^/K^+^-ATPase in the cortex to be different than the hippocampus. This point is discussed further in the Discussion section.

The fits to the observed Na^+^ and Ca^2+^ concentrations also require the expression level of NMDA and AMPA receptors in hippocampal astrocytes to be about 20% of cortical astrocytes—in line with the observed difference in expression levels of both receptor types in the two brain regions (Lalo et al., 2006; Ziemens et al., 2019). We also mimic the effect of NMDA receptor blocker 2-amino-5-phosphonovaleric acid (APV), AMPA receptor blocker 1,2,3,4-tetrahydro-6-nitro-2,3-dioxo-benzo[f]quinoxaline-7-sulfonamide (NBQX), and glutamate transporter blocker DL-threo-β-benzyloxyaspartate (TBOA) in our simulation to test the functionality of NMDA and AMPA receptors and glutamate transporters in the cortex and hippocampus. In the remaining of this section, we present the rate equations for Na^+^ and Ca^2+^ dynamics and some of the new fluxes included in our model. The remaining rate equations and fluxes are described in the Supplementary material. The parameters used in the Na^+^ and Ca^2+^ dynamics and initial conditions are listed Supplementary Tables S1–S3.

### Na^+^ dynamics

Intracellular Na^+^ is regulated by fluxes through Na^+^ leak channels (*J*_*Na*_), NKCC (*J*_*NKCC*_), *Na*^+^/*K*^+^-ATPase (*J*_*NaK*_), NCX (*J*_*NCX*_), glutamate transporters (*J*_*EAAT*_), NMDA receptors (*J*_*NMDA*_), and AMPA receptors (*J*_*AMPA*_). Thus, Na^+^ concentration in the ECS (Na_*o*_) and the astrocyte (Na_*a*_) are given by the following rate equations:

(1) $$\begin{array}{l} \begin{array}{l} \frac{dNa_{o}}{dt}=\frac{1}{VR_{sa}}(J_{Na}+3J_{NaK}-J_{NKCC}-(-3J_{NCX}+J_{NMDA_{Na}}\\ \;+J_{AMPA_{Na}}+3J_{GluT})\times \rho_{conv})+J_{NaNEtoSC}, \end{array} \end{array}$$

(2) $$\begin{array}{l} \begin{array}{l} \frac{dNa_{a}}{dt}=-J_{Na}-3J_{NaK}+J_{NKCC}-(3J_{NCX}-J_{NMDA_{Na}}-J_{AMPA_{Na}}-\\ \;3J_{GluT})\times \rho_{conv}. \end{array} \end{array}$$

VR_*sa*_ is the volume ratio of ECS to astrocyte. The number 3 in front of *J*_*NaK*_ indicates three Na^+^ leaving the astrocyte in exchange for two K^+^. The factor $\rho_{conv}=10^{3}\frac{A_{a}}{\left(F\times Vol_{a}\right)}$ converts $\frac{pA}{um^{2}}$ to $\frac{uM}{sec}$ where, A_*a*_ is area of the astrocyte, F is Faraday's constant, and Vol_*a*_ is astrocytic volume. Flux through NCX is adopted from Oschmann (2018) and is given as

(3) $$\begin{array}{l} \begin{array}{l} J_{NCX}=I_{NCXmax}H_{3}\left(Na_{o},K_{NCXmN}\right)H\left(Ca_{o},K_{NCXmC}\right)\times \\ \;\frac{\frac{Na_{a}^{3}}{Na_{o}^{3}}exp\left(\eta_{NCX}\times \frac{v_{a}}{v_{T}}\right)-\frac{Ca_{a}}{Ca_{o}}exp\left(\left(\eta_{NCX}-1\right)\times \frac{v_{a}}{v_{T}}\right)}{1+k_{sat}exp\left(\left(\eta_{NCX}-1\right)\times \frac{v_{a}}{v_{T}}\right)}, \end{array} \end{array}$$

Where H_*n*_(X, K) = $\frac{X^{n}}{K^{n}+X^{n}}$ and v_*T*_ = $\frac{RT}{F}$. R, T, F and v_*a*_ represent universal gas constant, temperature, Faraday's constant, and membrane potential of the astrocyte, respectively. I_*NCX*_*max*__ is the maximum current through NCX, which is adjusted to match our experimental results. K_*NCXmN*_, and K_*NCXmC*_ are the binding affinities of Na^+^ and Ca^2+^ to NCX, respectively. The energy barrier η_*NCX*_ determines the exchanger's reliance on the membrane voltage. k_*sat*_ assures that the current strength is saturated at high negative potentials. NCX imports 3 Na^+^ ions in exchange for one Ca^2+^ ion in the forward mode and vice versa in the reverse mode.

We model the current through glutamate transporters using the equations described in Oschmann (2018) as follows

(4) $$\begin{array}{l} \begin{array}{l} J_{GluT}=I_{GluTmax}H\left(K_{a},K_{GluTmK}\right)H_{3}\left(Na_{o},K_{GluTmN}\right)H\left(glu,K_{GluTmK}\right). \end{array} \end{array}$$

The maximum current through the transporters is represented by I_*GluTmax*_. To replicate our experimental results, the maximum transport current is changed from Oschmann (2018). The half-saturation constants for glutamate, Na^+^, and K^+^ are represented by K_*GluTmK*_, K_*GluTmN*_, and K_*GluTmg*_, respectively.

The net current through NMDA receptors is calculated using the equation

(5) $$\begin{array}{l} J_{NMDA}=J_{NMDA_{Na}}-J_{NMDA_{K}}+J_{NMDA_{Ca}}. \end{array}$$

The individual components are given as

(6) $$\begin{array}{l} J_{NMDA_{i}}=\frac{I_{INMDA_{max}}}{1.5}O_{NMDA}S\left(v_{a},b_{1},b_{2}\right)\left(v_{a}-E_{i}\right). \end{array}$$

Where S(v_*a*_, b_1_, b_2_) = $\frac{b1}{1+exp\left(b2\times v_{a}\right)}$ and *i* refers to Na^+^, K^+^ or Ca^2+^. E_*i*_ and I_*NMDA*_*max*__ is the reversal potential for Na^+^, K^+^ or Ca^2+^ current through NMDA receptors and maximum current through NMDA receptors, respectively. O_*NMDA*_ is the opening probability of NMDA receptor and is calculated as described in the Results section. The parameters b_1_ and b_2_ are obtained as explained in the Results section. E_*i*_ is calculated as

(7) $$\begin{array}{l} \begin{array}{l} E_{i}=\frac{RT}{z_{i}F}log\left(\frac{i_{o}}{i_{a}}\right), \end{array} \end{array}$$

Where z_*i*_, i_*o*_, and i_*a*_ represents the valency, extracellular, and intracellular concentration of the ion. The net current through AMPA receptors is modeled as

(8) $$\begin{array}{l} J_{AMPA}=J_{AMPA_{Na}}-J_{AMPA_{K}}, \end{array}$$

with the individual components given by

(9) $$\begin{array}{l} \begin{array}{l} J_{AMPA_{j}}=\frac{I_{AMPA_{max}}}{1.5}O_{AMPA}S\left(v_{a},b_{1},b_{2}\right)\left(v_{a}-E_{i}\right). \end{array} \end{array}$$

Where i here refers to Na^+^ or K^+^ passing through AMPA receptors. The open probability of AMPA receptor O_*AMPA*_ is calculated as described in the Results section.

### Ca^2+^ dynamics

Ca^2+^ concentration in the astrocyte is controlled by Ca^2+^ release from the ER through IP_3_Rs (*J*_*IP*_3__), SERCA (*J*_*pump*_), leak from the ER (*J*_*ERleak*_), influx across plasma membrane through TRPV4 channels (*J*_*TRPV*_), NCX (*J*_*NCX*_), and NMDA receptors (*J*_*NMDA*_*Ca*__). Accordingly, Ca_*a*_ is modeled with the following rate equation.

(10) $$\begin{array}{r} \begin{array}{l} \frac{dCa_{a}}{dt}=B_{cyt}\left(J_{IP3}-J_{pump}+J_{ERleak}-\frac{J_{TRPV}}{r_{buff}}\right)+\left(J_{NCX}+J_{NMDA_{Ca}}\right)\\ \times \rho_{conv}. \end{array} \end{array}$$

Here, B_*cyt*_ represents fast Ca^2+^ buffering and r_*buff*_ is the rate of Ca^2+^ buffering in astrocytic endfeet compared to the cell body. It should be noted that our simulations are not based on geometric model and assumes the astrocyte to be a point cell. However, following the approach in Kenny et al. (2018a,b), we assume the Ca^2+^ buffering capacity to be heterogeneous across the cell such that channels/receptors in different compartments contribute differently to the average free cytosolic Ca^2+^ concentration.

### Numerical methods

The rate equations are solved in Fortran 90 using Euler method with a time step of 0.1 μs. The system of equations is allowed to reach steady state before applying glutamate or NMDA. Data is visualized using MATLAB. Codes reproducing main results are available from authors upon request.

### Animal procedures

All animal procedures reported in this study were carried out in accordance with the institutional guidelines of the Heinrich Heine University Düsseldorf, as well as the European Community Council Directive (2010/63/EU) and were communicated to and approved by the animal welfare office of the animal care and use facility of the Heinrich Heine University Düsseldorf (institutional act number: O52/05). For the preparation of organotypic tissue slice cultures, wild-type Balb/C mice of both genders at postnatal days (P)6-8 were quickly decapitated and their brains rapidly removed. In accordance with the German Welfare Act (TSchG; Section Discussion, paragraph 3), no additional approval for post-mortem removal of brain tissue was necessary.

#### Preparation of organotypic brain tissue slices

Directly after dissection, brains were trimmed and placed in ice cold artificial cerebrospinal fluid (aCSF) containing (in mM): 130 NaCl, 2.5 KCl, 1.25 NaH_2_PO_4_, 26 NaHCO_3_, 2 CaCl_2_, 1 MgCl_2_, and 10 glucose; pH 7.4, bubbled with carbogen (95% O_2_, 5% CO_2_). They were cut into 250 μm thick parasaggital slices containing the neocortex and hippocampus using a vibratome (HM650V, Microtome, Thermo Fisher Scientific, Waltham, MA, USA). Slices were washed five times with acidified Hank's Balanced Salt Solution (Sigma-Aldrich, Munich, Germany), transferred onto Biopore membranes (Millicell standing insert, Merck Millipore, Burlington, VT, USA) and kept in an incubator at the interface between culture medium and humidified air containing 5% CO_2_ until used for experiments (Stoppini et al., 1991).

Experiments were performed in layers II/III of the somatosensory cortex and the CA1 region of the hippocampus at room temperature. During experiments, slices were constantly superfused with aCSF at a prefusion speed of 2–2.5 ml/min. All chemicals were purchased from Merck/Sigma-Aldrich (St. Louis, MO, USA) or AppliChem (Darmstadt, Germany) except for (2R)-amino-5-phosphonovaleric acid (APV), which was purchased from Cayman Chemical (Ann Arbor, Michigan, USA).

### Experimental determination of changes in intracellular ATP

Changes in intracellular ATP levels were determined in organotypic slice cultures using the genetically encoded FRET-based nanosensor ATeam1.03^YEMK^ (“ATeam”) (Imamura et al., 2009). After 1–3 days *in vitro*, 0.5 μl of a vector (AAV 5/2) carrying the code for ATeam under the astrocyte-specific promoter GFAP was applied on top of the tissue as described before (Lerchundi et al., 2019). Slices were maintained in the incubator for at least 10 days before being used for experiments. Transduced slices were imaged using an epifluorescence microscope (Nikon Eclipse FN-I, Nikon GmbH Europe, Düsseldorf, Germany) equipped with an Achroplan 40x objective (water immersion, N.A. 0.8; Nikon, Tokyo, Japan). ATeam was excited using a Poly-V monochromator (Thermo Fisher Scientific/FEI, Planegg, Germany) at 435 nm and images were taken at a frequency 0.5 Hz with a CMOS camera (Orca 4 LT Plus, Hamamatsu Photonics, Herrsching, Germany). Fluorescence emission was split at 500 nm (WVIEW GEMINI optic system; Hamamatsu Photonics, Herrschin, Germany) onto two bandpass filters (483/32: imaging of eCFP/donor, 542/27: imaging of Venus/acceptor). Fluorescence was collected from regions of interest (ROIs) manually drawn around cell bodies and the fluorescence ratio (Venus/eCFP) was calculated for individual ROIs. Subsequent analysis was performed offline using OriginPro2021 Software (OriginLab Corporation, Northhampton, MA, USA). Changes in the Venus/eCFP fluorescence ratio were normalized to the baseline and are given as percentage change thereof [ATeam ratio (%)]. Experimental data are presented in Tukey box-and-whisker plots. Lines represent the medians, squares the mean values, boxes the IQ50s and whiskers the standard deviations. Data were statistically analyzed by one-way ANOVA and *post hoc* Bonferoni test. *P* represents error probability, * 0.01 ≤ *P* ≤ 0.05, ** 0.001 ≤ *P* ≤ 0.01, *** < 0.001.

## Results

### Modeling astrocytic NMDA receptors

**Figure 2:**
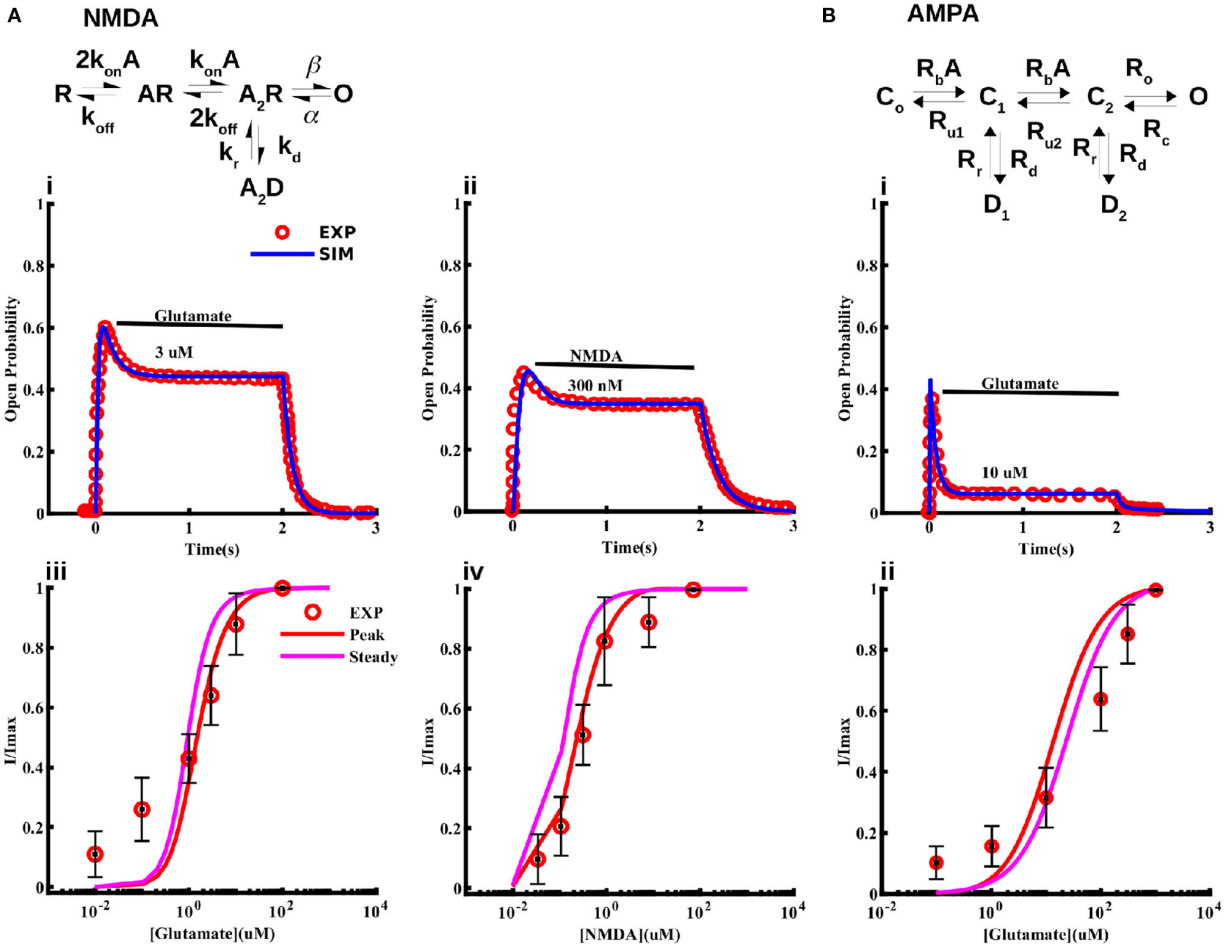
Models for NMDA and AMPA receptors in cortical astrocytes. Schematic of the 5-state model used for the NMDA receptor **(A)** and 6-state model used for the AMPA receptor **(B)**. The model fits to current traces from NMDA receptors in cortical astrocytes at different concentrations of glutamate **(Ai, Aiii)** and NMDA **(Aii, Aiv)**. In Ai and Aii 3 μM glutamate and 300 nM NMDA was applied for 2 s, respectively. **(Bi)** Sample trace from AMPA receptors given by the model and observed experimentally in response to 10 μM glutamate application for 2 s, and **(Bii)** normalized current through AMPA receptors as a function of glutamate concentration. In **(Aiii, Aiv, Bii)** we show both the peak and steady state values of the current from the model. Experimental data shown for comparison is extracted from Lalo et al. (2006).

As noted above, strong experimental evidence indicates the expression of NMDA and AMPA receptors in cortical astrocytes that can be activated by NMDA and glutamate, and contribute significantly to *Na*^+^ and Ca^2+^ signaling (Schipke et al., 2001; Lalo et al., 2006; Ziemens et al., 2019). While several single channel models for the gating of NMDA receptors exist, none of them is based on data from astrocytes. Thus, we first model the kinetics of NMDA receptors in cortical astrocytes. We consider the 5-state model first proposed for neuronal NMDA receptors in Clements and Westbrook (1991) and Lester and Jahr (1992) (Figure 2). According to this model, the receptor can be in agonist (A)-free resting state (R), one A-bound (AR), two agonist molecules-bound (A_2_R), a desensitize state (A_2_D), and an open state (O). We fit this model to current traces obtained at different concentrations of glutamate and NMDA reported in Lalo et al. (2006) taking the transition rates between different states as free parameters and using the least-squares fitting method (Figure 2). Sample fits to normalized time traces (or open probability) at 3 μM glutamate and 300 nM NMDA for 2 s are shown in Figure 2Ai,ii. Peak and steady state values of current at different concentrations of glutamate and NMDA are shown in Figure 2Aiii,iv, respectively. We also show the experimental values observed in Lalo et al. (2006) for comparison.

### Modeling astrocytic AMPA receptors

For AMPA receptors, we adopt the six-state model from Patneau and Mayer (1991) and Jonas et al. (1993) and fit it to current time-traces obtained from AMPA receptors in cortical astrocytes at different glutamate concentrations reported in Lalo et al. (2006) (Figure 2). The model has three closed states C_0_, C_1_, and C_2_ with no, one, and two ligands bound; two desensitized states D_1_ and D_2_ with one and two ligands bound; and one open state O. As in the case of NMDA receptors, all transition rates are taken as free parameters. We used this model to fit the data in Lalo et al. (2006) to find the parameters for the open probability of the AMPA receptor. Sample fit to a current trace in response to 10 μM glutamate for 2 s is shown in Figure 2Bi. Normalized peak and steady state current as functions of glutamate are shown in Figure 2Bii. Experimentally observed values from Lalo et al. (2006) are also shown for comparison.

Rate equations modeling the kinetics of NMDA and AMPA receptors are given in the Supplementary material. Parameters given by the fits are listed in Supplementary Tables S4, S5.

#### Current-voltage relationship

Lalo et al. (2006) also observed that, unlike neuronal NMDA receptors, astrocytic NMDA receptors are insensitive to magnesium ions. Thus, we modify the equation typically used for modeling the I–V relation of neuronal NMDA receptors by removing the magnesium dependence. Specifically, we use the following I–V relation.

(11) $$\begin{array}{l} \begin{array}{l} \frac{I_{v}}{I_{max}}=S\left(v_{a},b_{1},b_{2}\right)v_{a}, \end{array} \end{array}$$

**Figure 3:**
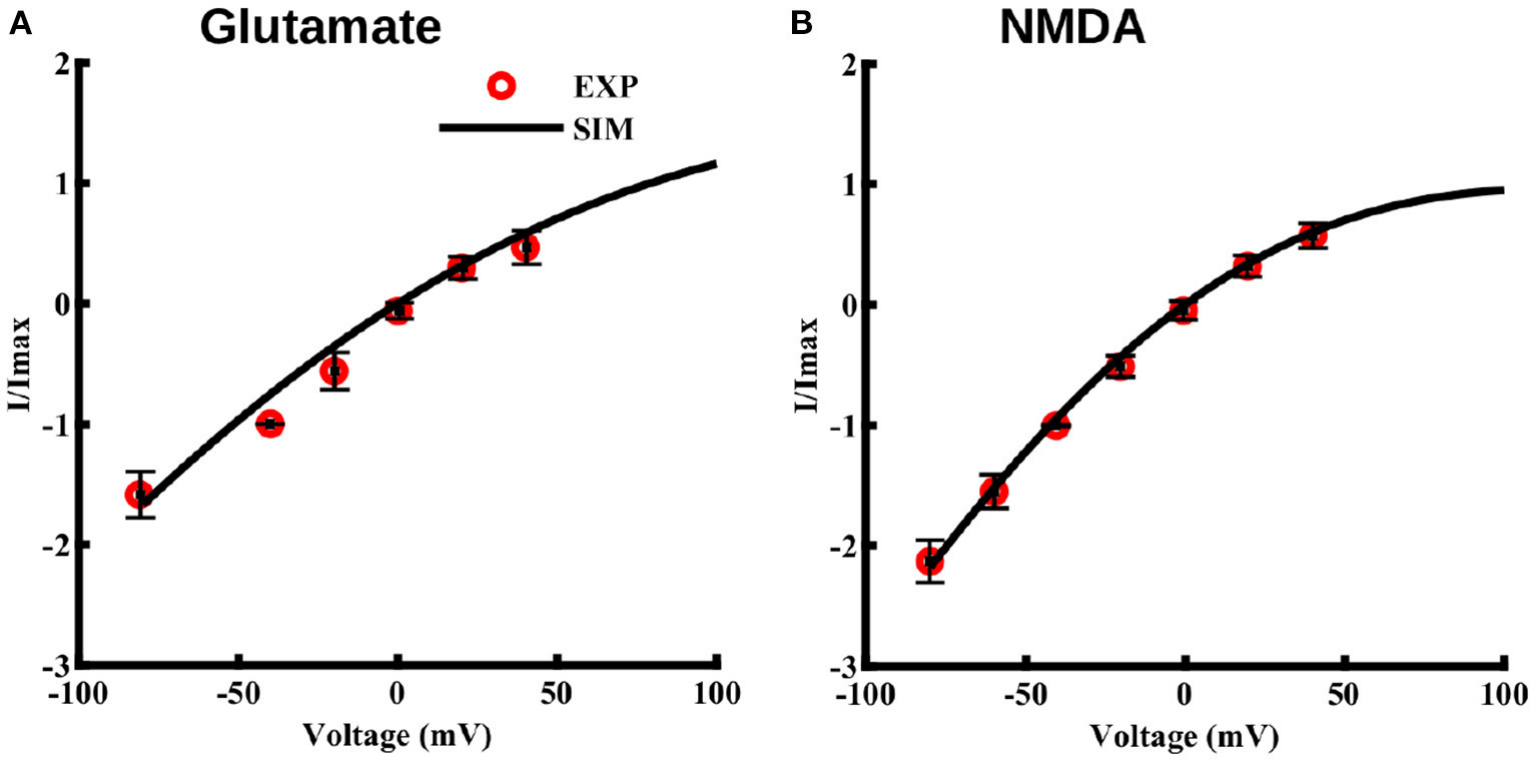
Current-voltage relationship for NMDA and AMPA receptors in cortical astrocytes in the presence of glutamate and for NMDA receptors in presence of NMDA. I–V curve in the presence of 10 μM glutamate **(A)** and 10 μM NMDA **(B)**, both applied for 2 s at different holding potentials. The values of current are normalized with respect to the current obtained at –40 mV. Solid lines represent model fits and circles represent the experimental values from 11 independent experiments from Lalo et al. (2006).

Where b_1_ and b_2_ are the free parameters obtained from the fit to the I–V values observed in Lalo et al. (2006). I_*max*_ represents the current obtained at –40 mV. Fits to maximum current through NMDA receptors due to 10 μM glutamate and 10 μM NMDA, both applied for 2 s at different voltage values, are shown in Figures 3A, B, respectively. Note that this voltage-dependence is incorporated in the current equation for NMDA (Equation 6) and AMPA (Equation 9) receptors.

### Different expression levels of NMDA and AMPA receptors lead to heterogeneous activity-evoked Na_*a*_ transients between the cortex and hippocampus

As noted above, there are two main differences in the models for cortical and hippocampal astrocytes: (1) the fits to the observed Na_*a*_ and Ca_*a*_ require the expression level of NMDA and AMPA receptors in cortical astrocytes to be five times higher than hippocampal astrocytes, and (2) to match the observed Na_*a*_ in the resting state (where we assume that the ligand concentration is 0 μM in the model) (Ziemens et al., 2019), we considered the peak pumping capacity of Na^+^/K^+^-ATPase in cortical astrocytes to be slightly lower than hippocampal astrocytes. As discussed in the Materials and Methods section, in the presence of ligand Na^+^/K^+^-ATPase is higher in the cortex to pump out the additional Na^+^ brought in by NMDA and AMPA receptors. As we will see below, the later result comes out naturally of the model without changing any parameter.

**Figure 4:**
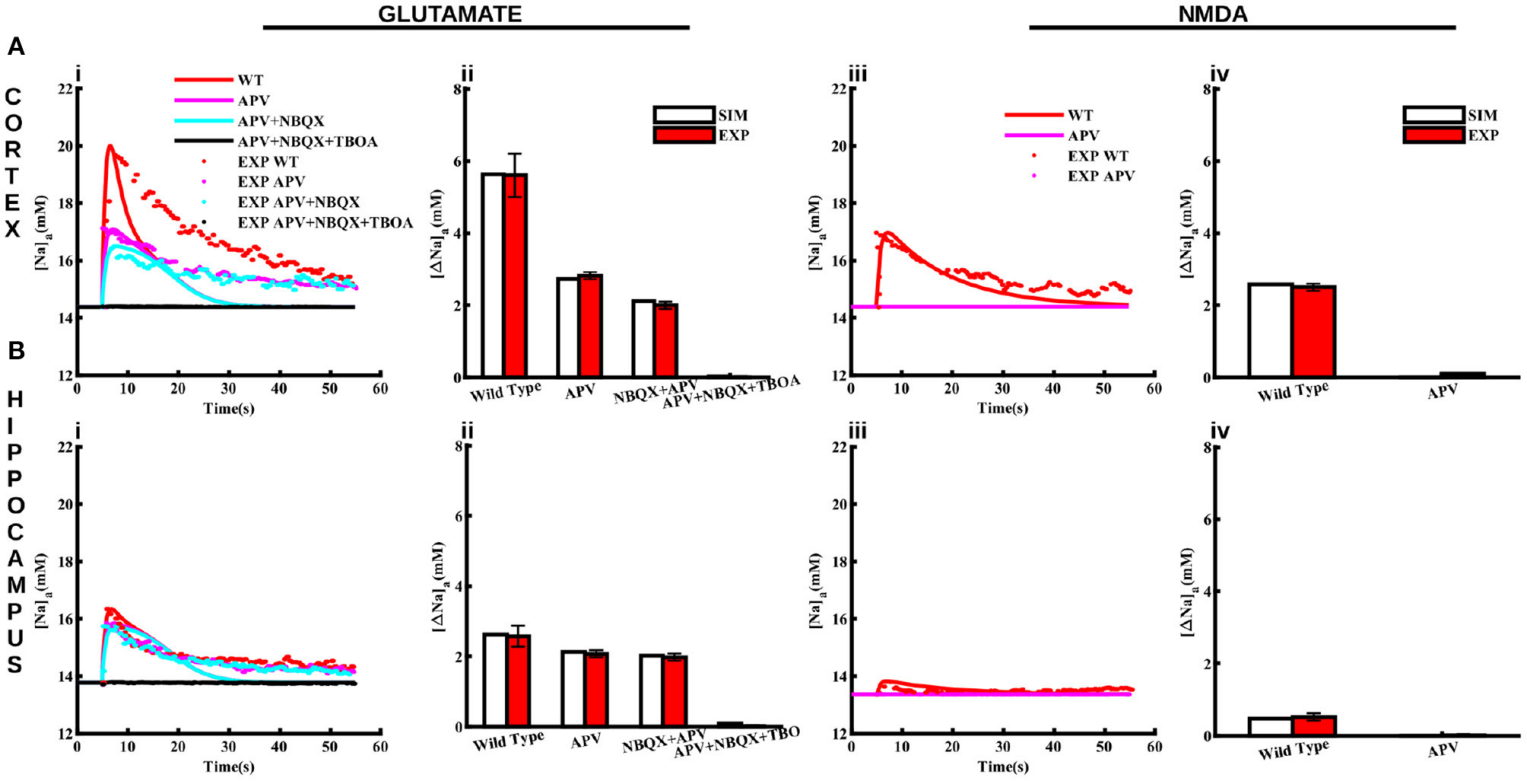
Pharmacological properties of glutamate and NMDA-induced Na_*a*_ transients in cortical and hippocampal astrocytes. **(A)** Changes in Na_*a*_ in the cortical astrocytes due to 1 mM glutamate applied for 100 ms **(Ai)** and 0.5 mM NMDA applied for 50 ms **(Aiii)** in control conditions (WT) and in the presence of APV, NBQX, and TBOA. The bar plots in **(Aii, Aiv)** represent the peak changes in Na_*a*_ estimated from the traces in **(Ai, Aiii)**, respectively. **(B)** Same as in **(A)** but for hippocampal astrocytes. Solid lines represent model fits whereas markers are experimental results adapted from Ziemens et al. (2019) for comparison.

The above two changes result in Na_*a*_ increases in response to glutamate (1 mM for 100 ms) (Figures 4Ai, Bi) and NMDA (0.5 mM for 0.5 s) (Figures 4Aiii, Biii) application that resemble those observed experimentally both in cortical and hippocampal astrocytes. The discrepancy between the decay phase of the traces from the model and experiment could be due to two factors. First, the decay time scale of the agonist in the tissue could be different than that used in the model. This time scale could also be different between the two brain regions. Second, the activity-evoked drop in the ATP level will decrease the peak capacity of Na^+^/K^+^-ATPase, leading to slower recovery of the Na^+^ gradient. Furthermore, as we will see later, the ATP consumption in the two brain regions is different, which will affect Na^+^/K^+^-ATPase differently, causing different decay rates of Na_*a*_. As a proof of concept, we show that fits to the decay phase of the time traces improve when the decay time scale of the agonist in the two regions is assumed to be different (Supplementary Figure S1). However, we believe that the slower and heterogeneous decay of Na_*a*_ would result naturally once the exact ATP homeostasis and activity-dependent decrease in Na^+^/K^+^-ATPase is incorporated in the model, which is beyond the scope of this study. Thus, we use the same decay rate for the agonist in both regions for the rest of the paper.

In addition to control conditions, the model also closely reproduces Na_*a*_ changes in response to glutamate and NMDA application in the presence of NMDA receptor blocker (APV), AMPA receptor blocker (NBQX), and glutamate transporters blocker (TBOA) as shown in Figure 4. Application of 1 mM glutamate increases Na_*a*_ in cortical and hippocampal astrocytes by 5.6 mM Figure 4Aii and 2.6 mM Figure 4Bii, respectively. Na_*a*_ amplitude drops to < 3 mM in the presence of APV in the cortex. However, Na_*a*_ amplitude decreases moderately in the hippocampal astrocytes due to APV. The addition of NBQX decreases Na_*a*_ further in both brain regions. Na_*a*_ drops to base level in the presence of APV, NBQX, and TBOA. The change in Na_*a*_ in response to 0.5 mM NMDA in both regions is significantly smaller (2.5 mM in cortex and ~0.5 mM in hippocampus) as compared to that in response to glutamate, which vanishes in the presence of APV (Figures 4Aiv, Biv). Overall, this data shows that our model not only reproduces changes in Na_*a*_ due to glutamate and NMDA in cortical and hippocampal astrocytes in control conditions but also in the presence of different pharmacological agents. Our results further show that in cortex, NMDA receptors and glutamate transporters play a major role in Na_*a*_ changes. In hippocampus, on the other hand, Na_*a*_ increase mostly occurs due to glutamate transporters.

### NMDA-mediated Na^+^ influx causes NCX to operate in reverse mode in cortical astrocytes

**Figure 5:**
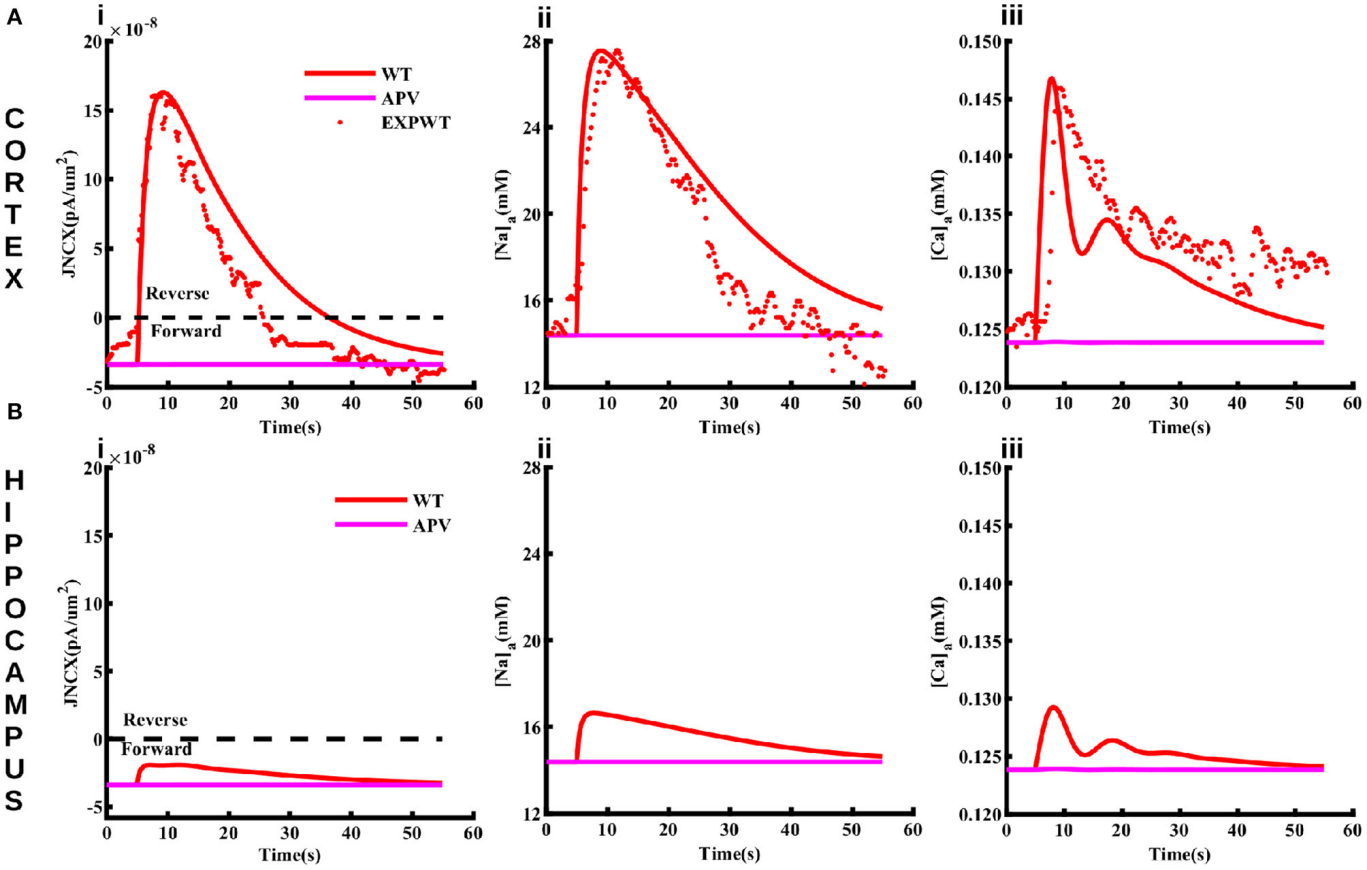
The function of NCX differs in cortical and hippocampal astrocytes. Flux through NCX **(Ai)**, Na_*a*_ **(Aii)**, and Ca_*a*_ **(Aiii)** in cortical astrocyte in response to 4 mM NMDA (model-equivalent of 50 mM in the iontophoresis pipette) applied for 0.5 ms. The solid and markers represent simulated and experimental values, respectively. The values above (positive) and below (negative) the black line in **(Ai)** represent the reverse and forward modes of NCX. Blocking NMDA receptors using APV causes NCX to operate in the forward mode and suppresses the rise in Na_*a*_ and Ca_*a*_ (pink lines) in cortical astrocytes. **(B)** same as in **(A)** but for hippocampal astrocytes.

NCX plays a major role in shaping Na_*a*_ by mediating Na^+^ entry in the forward mode and extruding Na^+^ in the reverse mode. In the reverse mode, NCX acts as a dynamic translator of astrocyte Na^+^ signals by converting them into influx of Ca^2+^ from the ECS (Blaustein and Lederer, 1999; Parpura et al., 2016; Gerkau et al., 2018; Jackson and Robinson, 2018; Rose et al., 2020). Here we investigate how NCX activity differs between cortical and hippocampal astrocytes upon neuronal activity. To preclude an effect of metabotropic glutamate receptors on Ca^2+^, we administer 4 mM NMDA (model-equivalent of 50 mM in the iontophoresis pipette) for 0.5 ms to the astrocyte and plot the current through NCX, Na_*a*_, and Ca_*a*_ in both brain regions (Figure 5). In the cortex, the large Na^+^ influx through NMDA receptors (Figure 5Aii) drives NCX into the reverse mode (above the black line in Figure 5Ai) where Ca^2+^ enters and Na^+^ leaves the astrocyte. The flux through NCX from the model also closely matches the values estimated experimentally (Ziemens et al., 2019). Both the model and experiment show that Na_*a*_ rises by more that 13 mM in cortical astrocyte (Figure 5Aii), driving NCX into reverse mode. As a result, Ca_*a*_ in the cortex also increases by more than 20 nM (Figure 5Aiii). Our model also predicts that due to the negligible Na^+^ influx through NMDA receptors in the hippocampus (Figure 5Bii), the flux through NCX is significantly smaller (Figure 5Bi), leading to a negligible change in Ca_*a*_ (Figure 5Biii). Furthermore, NCX operates in the forward mode (below the black line in Figure 5Ai) where Ca^2+^ leaves and Na^+^ enters the astrocyte. It is important to note that the Ca^2+^ rise in cortical astrocyte in these simulations is independent of the IP_3_ pathway (that is, the Ca^2+^ release from the intracellular stores does not contribute). Thus, these Ca^2+^transients will be most likely restricted to astrocytic processes as reported in Ziemens et al. (2019).

### Metabolic stress leaves cortical astrocytes more prone to Na^+^ and Ca^2+^ overload as compared to hippocampal astrocytes

Overall, the above results show that our model closely reproduces several observations about Na^+^ and Ca^2+^ homeostasis in astrocytes from cortex and hippocampus. Next, we use the model to predict how the heterogeneous Na^+^ and Ca^2+^ dynamics would affect other cell functions. We are specifically interested in the differences in astrocytic response to metabolic stress such as ischemic stroke in these two brain regions where cells are exposed to significantly higher levels of glutamate and the activity of Na^+^/K^+^-ATPase is compromised (Hansen and Zeuthen, 1981; Dreier, 2011; Ayata and Lauritzen, 2015; Enger et al., 2015; Hu and Song, 2017; Murata et al., 2020; Andrew et al., 2022). Accordingly, we simulate astrocytic Na^+^ and Ca^2+^ in response to 1 mM glutamate applied for 100 ms under two scenarios: (1) wild-type or control condition where the Na^+^/K^+^ pump functions normally and (2) a 20 s long metabolic stress. The metabolic stress is simulated by decreasing the maximum Na^+^/K^+^-ATPase capacity by 50% of its normal value. To test the effect of heterogeneity in the expression levels of NMDA receptors, we also repeat the simulations under metabolic stress in the presence of APV. It should be noted that the main conclusions remain the same if the duration of the metabolic stress is extended or the maximum Na^+^/K^+^-ATPase capacity is decreased further.

**Figure 6:**
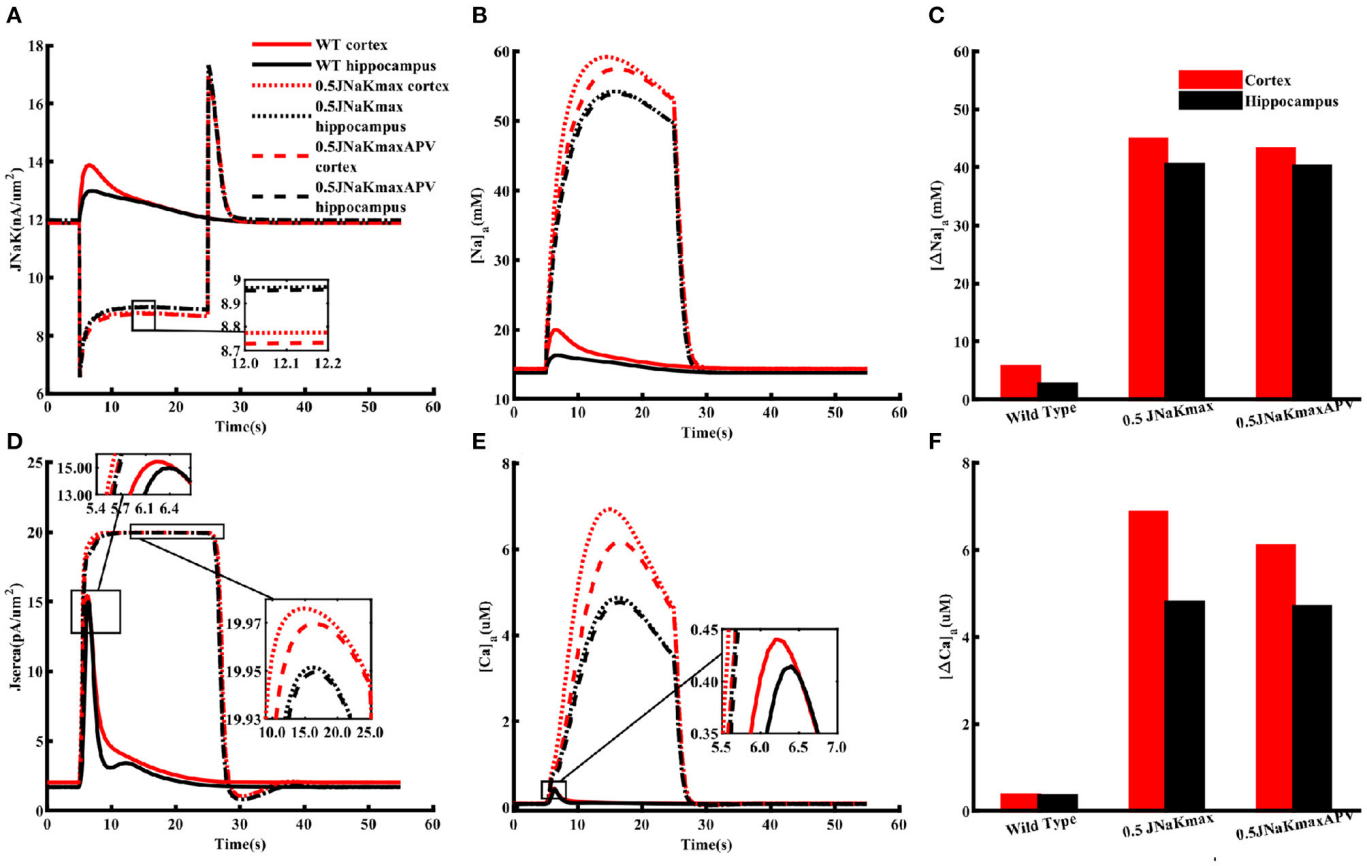
Metabolic stress leaves cortical astrocytes more prone to Na^+^ and Ca^2+^ overload as compared to hippocampal astrocytes. Flux through Na^+^/K^+^ pumps **(A)** and SERCA **(D)** under normal (WT) condition (solid lines), 20 s long metabolic stress where the peak capacity of Na^+^/K^+^-ATPase is reduced by 50% (dotted lines), and 20 s long metabolic stress in the presence of APV (dashed lines) in cortical (red) and hippocampal (black) astrocytes when both are exposed to 1 mM glutamate for 10 ms. **(B)** Na_*a*_ and **(E)** Ca_*a*_ from the simulation in **(A)**. Peak changes in Na_*a*_ **(C)** and Ca_*a*_ **(F)** during the simulations shown in **(A)**. Glutamate application and metabolic stress are initiated simultaneously. The insets are included to highlight the differences in various curves.

As shown in Figure 6A, flux mediated by Na^+^/K^+^-ATPase under control condition (solid lines) due to glutamate application is considerably larger in the cortex (red) as compared to the hippocampus (black). Metabolic stress (dotted lines) causes a sharp initial drop in Na^+^/K^+^-ATPase activity, which plateaus to a slightly lower value in the cortex. The flux overshoots to a larger value as compared to the control value when the energy supply is restored before reaching resting state. Although, we do not change the peak capacity of SERCA in these simulations, the change in Na^+^/K^+^-ATPase significantly increases the flux through SERCA in both the cortex and hippocampus (Figure 6D). Metabolic stress causes substantially larger increase in Na_*a*_ (Figures 6B, C) and Ca_*a*_ (Figures 6E, F) with the rise in the cortex significantly larger than the hippocampus. Na_*a*_ rises from ~ 14 to ~ 60 mM in the cortex and ~ 13 mM to ~ 54 mM in the hippocampus. Similarly, Ca_*a*_ reaches 7 μM in the cortex and 4.5 μM in the hippocampus. Blocking NMDA receptors with APV reduces the peak rise in both Na_*a*_ and Ca_*a*_ (dashed lines). Overall, these results show that metabolic stress leaves cortical astrocytes more prone to Na^+^ and Ca^2+^ overload as compared to hippocampal astrocytes. It should be noted that, in addition to the impaired Na^+^/K^+^-ATPase, metabolic stress also leads to a significant rise in the concentrations of glutamate and K^+^ in the ECS. Incorporating such effects in the model does not change the main conclusions from the model. Nevertheless, one would expect the Na^+^ and Ca^2+^ overload to be even more severe in the intact brain. Furthermore, due to the higher expression of NMDA and AMPA receptors the additional glutamate in the ECS would result in a higher Na^+^ accumulation in neocortical astrocytes as compared to the hippocampus.

### NMDA receptor lead to higher ATP consumption in cortical astrocytes

**Figure 7:**
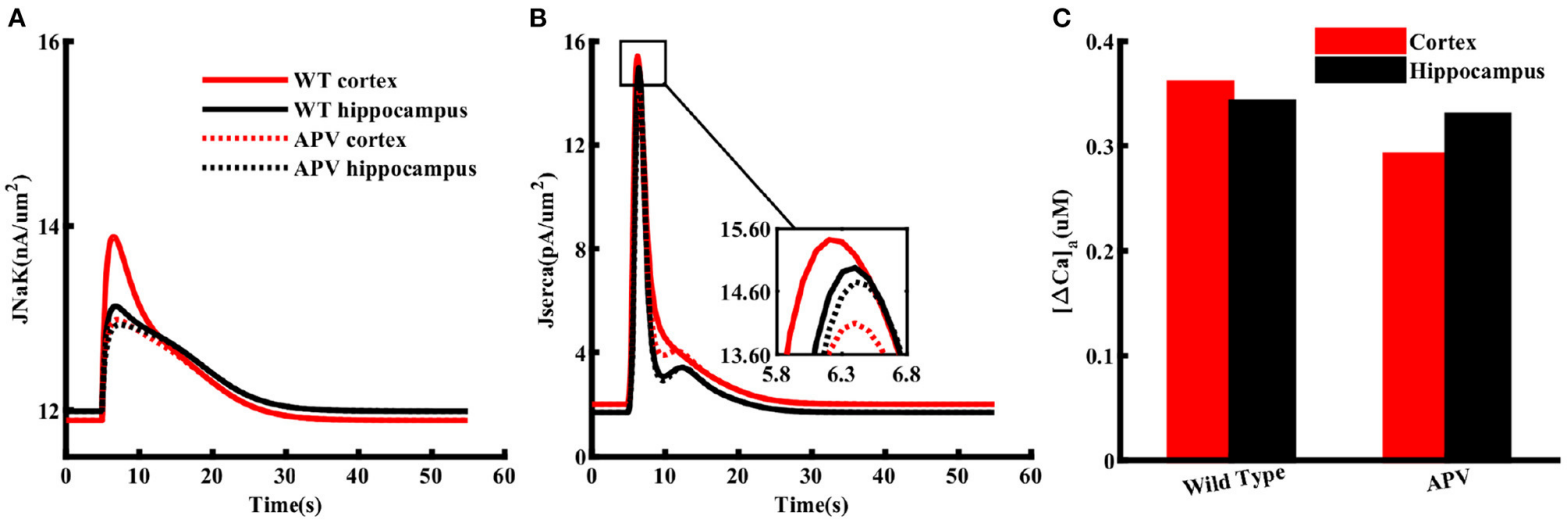
NMDA receptors cause higher ATP consumption in cortical astrocytes than those in the hippocampus. Flux through Na^+^/K^+^ pumps **(A)** and SERCA **(B)** in normal condition (solid lines) and in the presence of APV (dotted lines) in cortical (red) and hippocampal (black) astrocytes exposed to 1 mM glutamate for 100 ms. **(C)** Peak changes in Ca_*a*_ during the simulations shown in **(A)**.

The higher Na^+^/K^+^-ATPase activity in response to a glutamate pulse also indicates that the energy usage by cortical astrocytes should be larger than those in the hippocampus. It is also natural to assume that this difference in energy consumption results from the higher increase in Na^+^ due to higher expression of NMDA receptors in the cortex. To confirm this in the model, we mimicked the effect of APV by blocking NMDA receptors in astrocytes exposed to 1 mM glutamate for 100 ms. As clear from Figure 7A, flux through Na^+^/K^+^ pumps drop to similar levels in both brain regions in the presence of APV. The SERCA flux is also noticeably stronger in cortex as compared to hippocampus in control condition, which drops to the same peak value when APV is applied (Figure 7B). The dip in the SERCA flux during the decaying phase is due to the forward mode of NCX in the hippocampus in normal condition or in the presence of APV in both brain regions. For completion, we also show the peak change in Ca_*a*_ in both brain regions (Figure 7C). As expected, APV causes a higher drop in Ca_*a*_ in the cortex as compared to the hippocampus.

### Experimental determination of changes in intracellular ATP

**Figure 8:**
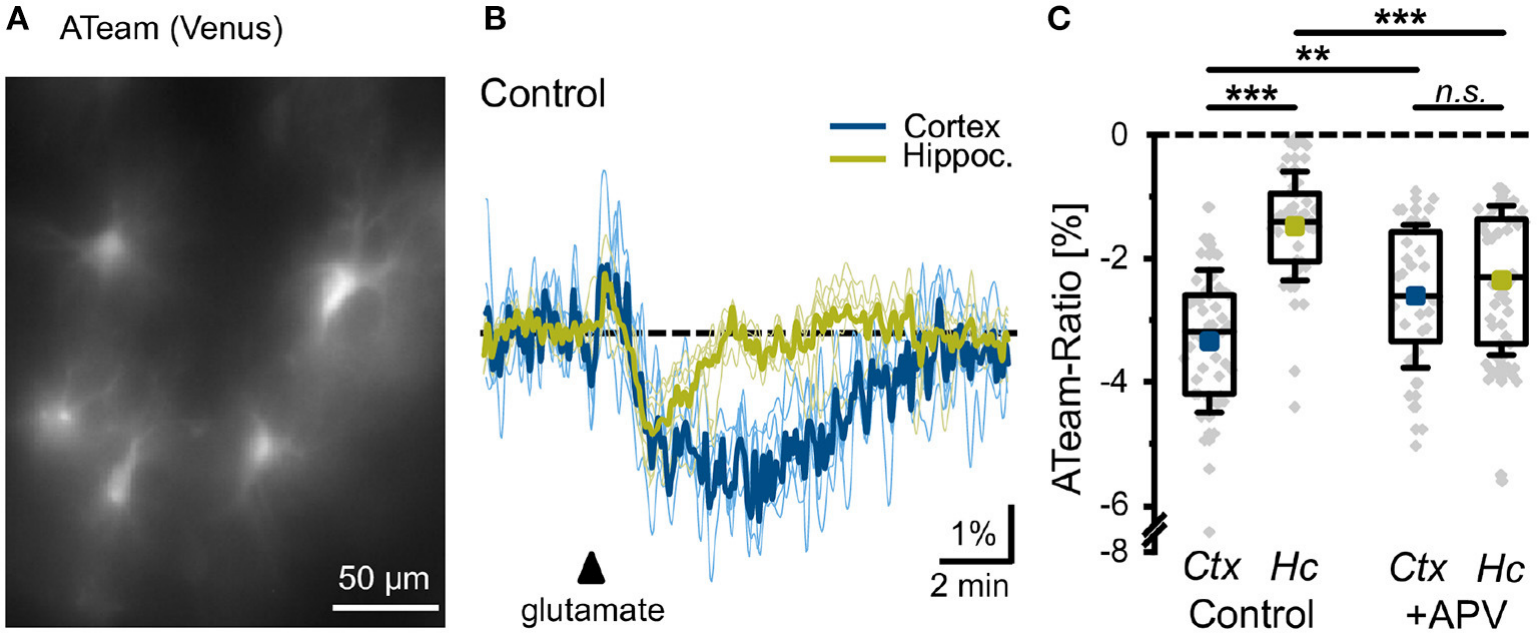
Glutamate-evoked changes in astrocytic ATP. **(A)** Image of the Venus fluorescence of ATeam expressed under the GFAP promoter in an organotypic cultured slice of the mouse neocortex. **(B)** Changes in the ATeam ratio of two individual astrocytes in the somatosensory cortex (black trace) and the hippocampus (gray trace) evoked by a 10 s glutamate bath application (arrowhead). Traces were smoothed for illustration purposes using an FFT filter 3. **(C)** Box plots of peak changes in astrocytic ATeam ratio upon glutamate application in the neocortex (Ctx) and hippocampus (Hc) under control conditions and in the presence of the NMDA-receptor blocker APV. Shown are individual data points (gray diamonds), means (squares), medians (horizontal lines), IQ50 (boxes), and SD (whiskers). **p < 0.01, ***p < 0.001, and n.s., not significant (*P* = 0.35).

In order to validate the prediction from our model, we studied the effect of glutamate on astrocytic ATP levels experimentally. To this end, we expressed the nanosensor ATeam1.03^YEMK^ (“ATeam”) in organotypic tissue slice cultures of the neocortex and hippocampus (Imamura et al., 2009; Lerchundi et al., 2019) (Figure 8A). Perfusion of slices with aCSF containing 1 mM glutamate for 10 s caused a biphasic change in the ATeam ratio in both brain regions. It consisted of an initial brief increase in the ATeam ratio by about 1%, which was followed by a more pronounced and prolonged decrease (Figure 8B). In astrocytes of neocortical layers II/III, the ATeam ratio transiently decreased by 3.3 ± 1.2% (*n* =46 cells, 8 tissue slices, 5 animals). The peak decline was reached at 3–4 min after the stimulation upon which the ATeam ratio recovered to the initial baseline level within another 4–5 min (Figures 8B, C). In astrocytes of the hippocampal CA1 area, the glutamate-induced decrease in the ATeam ratio was significantly smaller, amounting to only 1.5 ± 0.9% (*n* = 47/8/5; *p* = 1 × 10^−13^) (Figures 8B, C).

To study the involvement of NMDA receptors in the glutamate-induced changes in astrocytic ATP levels, we repeated the experiments in the presence of the NMDA receptor blocker APV (100 μM). In neocortical astrocytes, the amplitude of the glutamate-induced decrease in the ATeam ratio was significantly reduced to 2.6 ± 1.1% (*n* = 32/6/4; *p* = 0.0066), very close to the change in the hippocampus with APV (2.4 ± 1.2%; *n* = 54/8/5; *p* = 8 × 10^−5^) (Figure 8C). Notably, the latter was even larger as compared to control condition (1.5 ± 0.9%) (Figure 8C).

Taken together, these results indicate that glutamate results in an initial transient increase in astrocytic ATP, followed by a pronounced and longer-lasting decline. In neocortical astrocytes, the long-lasting decrease in the intracellular ATP concentration is reduced by blocking NMDA receptors. In hippocampal astrocytes, the glutamate-induced reduction in cellular ATP levels is significantly smaller in amplitude as compared to the neocortex, and is not reduced, but increased by NMDA receptor inhibition.

## Discussion

Emerging evidence shows that neuronal activity-related changes in Na_*a*_ represent a special form of astrocytic excitability, which is tightly coupled to all other major ions. Thus, a breakdown in astrocytic Na^+^ homeostasis leads to a secondary breakdown of other ion homeostases as well (Rose and Ransom, 1996; Somjen, 2004; Kelly et al., 2009; Langer and Rose, 2009; Attwell et al., 2010; Kirischuk et al., 2012; Parpura and Verkhratsky, 2012; Rose and Karus, 2013; Chatton et al., 2016; Rose and Verkhratsky, 2016; Gerkau et al., 2017, 2019; Rose et al., 2018a,b, 2020; Ziemens et al., 2019; Felix et al., 2020; Verkhratsky et al., 2020). Na_*a*_ also plays a key role in controlling neuronal excitotoxicity by facilitating the uptake of glutamate and GABA by astrocytes through Na^+^-dependent transporters (Kirischuk et al., 2016; Rose and Verkhratsky, 2016; Rose et al., 2018a). Furthermore, neurons strongly depend on the energy provided by astrocytes (Marcaggi and Attwell, 2004; Allaman et al., 2011; Gerkau et al., 2017), which is regulated by astrocytic Na^+^ (Chatton et al., 2016; Hernansanz-Agustín et al., 2020). For example, K^+^ released during high neuronal activity depolarizes astrocytes, activating inward Na^+^-bicarbonate co-transport, which stimulates glycolysis. High glycolytic activity leads to increased lactate production, which is transported to neurons through lactate shuttle. Similarly, glutamate released by glutamatergic neurons is buffered by astrocytes through glutamate transporters in Na^+^-dependent manner. Glutamate is then converted to glutamine, which is transported to neurons through the Na^+^-dependent transporter SN1 (see Gerkau et al., 2017 for more details). In summary, there is substantial evidence that astrocytic Na^+^ homeostasis is crucial to maintaining ionic and metabolic balance both in the astrocytes and surrounding neurons and for protection against excitotoxicity (Karus et al., 2015).

Despite playing these key roles in neuronal and astrocytic functions, the molecular and cellular mechanisms underlying Na^+^ homeostasis in astrocytes remain incompletely understood. Moreover, a biophysical model replicating the key observations about Na_*a*_ dynamics is missing. Similarly, to our knowledge, none of the existing models take into account the brain-specific differences in the Na^+^ and/or Ca^2+^ signaling in the astrocytes. Here we developed a detailed computational model that not only reproduces several observations about Na_*a*_ under different pharmacological conditions but also makes testable predictions about the downstream effects of changes in Na^+^ homeostasis in cortical and hippocampal astrocytes.

A key missing component in the existing models of astrocytic ion homeostasis is the fluxes through ionotropic glutamate receptors. Since cortical astrocytes are shown to express high levels of NMDA and AMPA receptors (Schipke et al., 2001; Lalo et al., 2006; Palygin et al., 2010), and all previous models of these two channel types are based on non-astrocytic data, we first modified the existing models so that they would mimic the observed gating behavior of these channels in astrocytes (Lalo et al., 2006). For both NMDA and AMPA receptors, the models closely fit the current time-traces at a given glutamate concentration (Figure 2), peak currents as a function of glutamate concentration (Figure 2), and peak currents as a function of membrane potential (Figure 3). The NMDA receptor model also provides a close fit to current through the channel at different NMDA concentrations (Figure 2).

The models for NMDA and AMPA receptors are added to a detailed model incorporating the dynamics of astrocytic membrane potential and concentrations of Na^+^, Ca^2+^, K^+^, and Cl^-^ in the astrocyte and ECS. The model also includes glutamate uptake through transporters, IP_3_ production through metabotropic glutamate receptors, and Ca^2+^ exchange with the ER. The resulting model closely fits the observed changes in Na_*a*_ under different pharmacological conditions and predicts that fitting the observed Na_*a*_ and Ca_*a*_ requires the expression level of NMDA and AMPA receptors in cortical astrocytes to be at least five times higher than hippocampal astrocytes (Ziemens et al., 2019).

Our model also predicts that under resting conditions where no agonist is present, the peak pumping rate of Na^+^/K^+^-ATPase is 3% lower in the cortex than hippocampus. This difference could be due to the heterogeneity in the expression of Na^+^/K^+^-ATPase across different brain regions (Blanco, 2005; Verkhratsky and Nedergaard, 2018; Murata et al., 2020). Astrocytes express a Na^+^/K^+^ complex containing α1 and α2 subunits where α1 is believed to set the baseline Na_*a*_ and α2 handles the Na^+^ load during periods of high neuronal activity (Pellerin and Magistretti, 1997; Zahler et al., 1997; Rose and Chatton, 2016). The expression of both subunits vary from one brain region to another, potentially leading to the differences in Na^+^/K^+^-ATPase in the resting state (Blanco, 2005; Murata et al., 2020). Specifically, a higher expression level of α1 subunit was reported in the hippocampus of neonatal mouse brain as compared to the cortex (Sundaram et al., 2019). The same study also reported a higher expression level of α2 subunit in the hippocampus of adult mouse brain. The lower expression level of α2 subunit in the cortex would also contribute to the higher Na_*a*_ accumulation during periods of high activity. This is why the amplitude of Na_*a*_ in the cortex is slightly higher than that in the hippocampus even when both NMDA and AMPA receptors are blocked (see Figures 4, 6).

Both the model and experimental results show that NMDA receptors and glutamate transporters are the major contributors to glutamate-induced Na_*a*_ increase in the cortex with minimal effect due to AMPA receptors (Figure 4). In the hippocampus, on the other hand, Na_*a*_ transients mostly arise due to influx through glutamate transporters. NMDA also evokes significant rise in Na_*a*_ in the cortex, which is blocked by NMDA receptor blocker APV. The NMDA-evoked rise in Na_*a*_ is largely absent in the hippocampus (Figure 4).

One of the key pathways coupling Na^+^ and Ca^2+^ homeostases in astrocytes and neurons is the Na^+^/Ca^2+^ exchanger, which imports three Na^+^ in exchange for one Ca^2+^ in forward mode (Blaustein and Lederer, 1999; Parpura et al., 2016). The model predicts that the NMDA receptor-mediated Na_*a*_ rise in the cortex drives NCX in the reverse mode, causing Ca^2+^ to flow inward and Na^+^ outward (Figure 5). This leads to a significant increase in Ca_*a*_ even in the absence of IP_3_ pathway as metabotropic glutamate receptors are not activated by NMDA. In hippocampal astrocytes, on the other hand, Ca^2+^ signaling due to NMDA-mediated current is minimal because of the negligible rise in Na_*a*_. This result is crucial as the localized rises in Ca^2+^ concentration in the processes of astrocytes have been correlated with the localized Na^+^ transients, which could serve to meet local metabolic demands without evoking global metabolic changes (Kirischuk et al., 2012; Boddum et al., 2016; Boscia et al., 2016; Parpura et al., 2016; Brazhe et al., 2018; Gerkau et al., 2018; Ziemens et al., 2019). It should be noted that NCX might switch to reverse mode in hippocampal astrocytes if stimulated by glutamate as the Na^+^ influx through glutamate transporters might raise Na_*a*_ high enough to switch the direction of NCX. Thus, our results show that the localized Na^+^ and Ca^2+^ signals would differ between different brain regions. Furthermore, the possibility of initiating global Na^+^ and Ca^2+^ waves would be higher in case of glutamate where the flux through glutamate transporters and IP_3_ pathway both are activated.

In addition to modulating ATP production through mitochondrial NCX, stimulating glycolysis, and glutamate-glutamine cycle, a rise in intracellular Na^+^ also comes with significant energy consumption. More than half of the energy used by neurons and astrocytes is consumed by various ion pumps to maintain and reestablish ion gradients (Erecińska and Silver, 1994; Somjen, 2004; Foo et al., 2012; Gerkau et al., 2017). Na^+^/K^+^-ATPase, in particular, has been estimated to consume nearly 50% of the cellular ATP (Astrup et al., 1981; Ames, 2000; Attwell and Laughlin, 2001; Foo et al., 2012). Thus, we investigated how a similar drop in the peak capacity of Na^+^/K^+^-ATPase would affect astrocytic function in cortex and hippocampus. Our model predicts that cortical astrocytes are prone to significantly higher Na^+^ and Ca^2+^ overload during metabolic stress than hippocampal astrocytes (Figure 6). Conversely, Na^+^/K^+^ pumps work harder to restore Na^+^ gradients across astrocytic membrane in the cortex under metabolic stress or when exposed to high concentrations of glutamate or NMDA (Figure 7). The model further predicts that the difference in ATP consumption results from the higher expression of NMDA receptors in the cortex. Thus, blocking NMDA receptors should restore ATP values in the cortex and hippocampus to similar values. Predictions from our model are validated by our experiments measuring ATP in astrocytes in control conditions and in the presence of NMDA receptor blocker APV (Figure 8).

To summarize, astrocytes display a remarkable morphological and functional heterogeneity (Matyash and Kettenmann, 2010; Verkhratsky and Nedergaard, 2018), which plays a crucial role in the pathophysiology of central nervous system (Pekny et al., 2016; Rodríguez-Arellano et al., 2016; Escartin et al., 2021; Poulot-Becq-Giraudon et al., 2022). Accordingly, a growing body of evidence shows that the effects of disrupted astrocytic function and morphologies on brain function are not uniform, but vary in a context- and brain region-specific manner (Pekny et al., 2016). An emerging area of interest is the disruption of neurotransmitter recycling in aging diseases, such as Alzheimer's, and its intimate link to impairments in astrocyte energy metabolism (Andersen et al., 2022). Furthermore, there is strong evidence that astrocytic neurotransmitter uptake and energy metabolism are tightly linked to their Na^+^ homeostasis (Langer et al., 2017; Rose et al., 2018a; Felix et al., 2020; Verkhratsky and Rose, 2020). In light of these observations, gaining a deeper insight into Na^+^ signaling and how it varies from one brain region to another is crucial to understanding the pathophysiological role of astrocytes. Here we developed the first computational model, based on extensive experimental data, that not only accounts for several observations about Na^+^ and Ca^2+^ homeostasis in astrocytes in the cortex and hippocampus, but also makes testable predictions. Specifically, our model predicted that the higher expression level of NMDA receptors should lead to higher ATP consumption in the cortex than the hippocampus, which was confirmed experimentally in organotypic slices from mice.

We remark that our model is a simple representation of a very complex reality. Several pathways that could affect Na^+^ and Ca^2+^ homeostases and their spatial variability throughout the astrocyte are omitted in the model. Similarly, how the heterogeneity in Na^+^ and Ca^2+^ homeostasis affect neurotransmitter uptake and ATP production in different brain regions remains the focus of our future research. Nevertheless, we believe that our model will prove a valuable tool in investigating neuron-astrocyte interaction and neurovascular coupling during normal and pathological brain function.

## Data availability statement

The original contributions presented in the study are included in the article/Supplementary material, further inquiries can be directed to the corresponding author.

## Ethics statement

The animal study was reviewed and approved by Animal Welfare Office of the animal care and use facility of the Heinrich 149 Heine University Düsseldorf (institutional act number: O52/05).

## Author contributions

GU and CRR contributed to conception and design of the study. PT and NP performed experiments and carried out statistical analysis. PT wrote the first draft of the manuscript. NP wrote sections of the manuscript. All authors contributed to manuscript revision, read, and approved the submitted version.
