# Supplementary material for: Modeling the heterogeneity of sodium and calcium homeostasis between cortical and hippocampal astrocytes and its impact on bioenergetics
Source: Front Cell Neurosci. 2023 Jan 30;17:1035553. doi: 10.3389/fncel.2023.1035553 (PMC9922870; doi:10.3389/fncel.2023.1035553)
Supplement: Supplementary file 1 [file Presentation_1.pdf]

Supporting Information Text:

Modeling the heterogeneity of sodium and calcium homeostasis between cortical and hippocampal astrocytes and its impact on bioenergetics

1 Model of NMDA receptor

The NMDA receptor can be in agonist A-free resting state (R), one A-bound (AR), two agonist molecules-bound (A_2R), a desensitize state (A_2D), and an open state (O). The probabilities of these states are given by the following rate equations:

$$\frac{dAR}{dt} = 2k_{on} \times A \times R + 2k_{off} \times A_2R - \big(k_{off} + k_{on} \times A\big) \times AR, \tag{1}$$

$$\frac{dA_2R}{dt} = k_{on} \times A \times AR + \alpha \times O + k_r \times A_2D - \big(2k_{off} + k_d + \beta\big) \times A_2R, \tag{2}$$

$$\frac{dO}{dt} = \beta \times A_2R - \alpha \times O, \tag{3}$$

$$\frac{dA_2D}{dt} = k_d \times A_2R - k_f \times A_2D. \tag{4}$$

The probability of state R is given by the conservation of probability i.e.

$$R = 1 - AR - A_2R - A_2D - O. \tag{5}$$

The open probability of NMDA receptor (O_{NMDA}) is equal to O.

2 Model of AMPA receptor

AMPA receptor can be in agonist (A)-free resting state (C_0), one A-bound (C_1), two A-bound (C_2), a desensitize state with one A-bound desensitize state (D_1), two A-bound desensitize state(D_2), and an open state(O). The probabilities of these states are given by the following rate equations:

$$\frac{dC_1}{dt} = R_b \times A \times C_0 - \big(R_{u1} + R_b \times A + R_d\big)C_1 + R_{u2} \times C_2 + R_r D_1, \tag{6}$$

$$\frac{dC_2}{dt} = R_b \times A \times C_1 + R_c \times O + R_r \times D_2 - (R_{u2} + R_o + R_d\big)C_2, \tag{7}$$

$$\frac{dD_1}{dt} = R_d \times C_1 - R_r \times D_1, \tag{8}$$

$$\frac{dD_2}{dt} = R_d \times C_2 - R_r \times D_2, \tag{9}$$

$$\frac{dO}{dt} = R_o \times C_2 - R_c \times O. \tag{10}$$

The probability of state C_0 is given by the conservation of probability, i.e.

$$C_0 = 1 - C_1 - C_2 - D_1 - D_2 - O. \tag{11}$$

The open probability of AMPA receptor (O_{AMPA}) is equal to O

3 Ion dynamics in ECS and astrocyte

K^+ concentration in the ECS (K^+) depends upon the fluxes of Na^+/K^+-ATPase pump, K^+ channels, co-transporter, inward rectified channels as well as K^+ exchange with the bath solution. The rate equation for extracellular K^+ concentration is

$$\frac{dK_o}{dt} = \frac{1}{VR_{sa}}\Big(J_K - 2J_{NaK} - J_{NKCC} - J_{KCC} + J_{BK} + \big(J_{NMDA_K} + J_{AMPA_K} + J_{GluT}\big) \times \rho_{conv}\Big) + J_{KNEtoSC}, \tag{12}$$

where VR_{sa} is the volume ratio between ECS to astrocytic region. The flux through the K^+ channel (μMs^{-1}) is

$$J_K = G_K\left(v - E_K\right). \tag{13}$$

G_K is whole-cell conductance of K^+, v is the membrane potential, and E_K is the reversal potential of K^+ channel. Nernst potential for the K^+ channel (mV) is

$$E_K = \frac{v_T}{z}\ln\left(\frac{K_o}{K_a}\right), \tag{14}$$

where $v_T = \frac{RT}{F}$.

Na^+/K^+-ATPase exchanges 3 Na^+ to exit the astrocyte for the influx of 2 K^+. The flux through the Na^+/K^+ pump (μMs^{-1}) is

$$J_{NaK} = J_{NaK_{max}} H_{1.5}\big(Na_a, K_{Na_a}\big) H\big(K_o, K_{K_o}\big). \tag{15}$$

$J_{NaK_{max}}$ is a maximum flux through Na^+/K^+ pumps.

One Na^+, one K^+, and 2 Cl^- ions move in inward direction through NKCC1. The flux through NKCC (μMs^{-1}) is

$$J_{NKCC1} = G_{NKCC1} v_T \ln\left(\frac{Na_o K_o Cl_o^2}{Na_a K_a Cl_a^2}\right). \tag{16}$$

G_{NKCC} is the whole-cell conductance of NKCC.

Cl^- and K^+ flux through the KCC channel (μMs^{-1}) :

$$J_{KCC1} = G_{KCC} v_T \ln\left(\frac{K_o Cl_o}{K_a Cl_a}\right). \tag{17}$$

NMDA receptor allows the flow ofNa^+ and Ca^{2+} into the cell and K^+ in the outward direction. The flux through NMDA receptors is

$$J_{NMDA} = J_{NMDA_{Na}} - J_{NMDA_K} + J_{NMDA_{Ca}}, \tag{18}$$

$$J_{NMDA_i} = \frac{I_{INMDAmax}}{1.5} O_{NMDA} S\big(v_a, b_1, b_2\big)\big(v - E_i\big). \tag{19}$$

E_i is the Nernst potential where i refers to Na^+, K^+ or Ca^{2+}. $I_{NMDAmax}$ is the maximum flux through NMDA receptors and O_{NMDA} is the opening probability of NMDA receptor.

The K^+ flux through AMPA receptor is

$$J_{AMPA} = J_{AMPA_{Na}} - J_{AMPA_K}, \tag{20}$$

$$J_{AMPA_{ii}} = \frac{I_{AMPA_{max}}}{1.5} O_{AMPA} S\big(v_a, b_1, b_2\big)\big(v - E_i\big). \quad (21)$$

Here i refers to Na^+ or K^+. $I_{AMPA_{max}}$ is the maximum flux through AMPA receptors and O_{AMPA} is the open probability of AMPA receptor.

K^+ flux due to glutamate transporters is

$$J_{GluT} = I_{GluTmax} H\big(K_a, K_{GluTmK}\big) H_3\big(Na_o, K_{GluTmN}\big) H\big(glu, K_{GluTmg}\big). \quad (22)$$

$I_{GluTmax}$ is the maximum current through glutamate transporters. K_{GluTmK}, K_{GluTmN}, and K_{GluTmg} represent the half-saturation constant for K^+, Na^+, and glutamate, respectively. ρ_{conv} converts pA/um^2 to uM/sec and is given as

$$\rho_{conv} = 10^3 \frac{A_a}{\big(F \times vol_a\big)}. \quad (23)$$

A_a is the area of the astrocyte, F is Faraday's constant, and vol_a is the astrocyte volume.

K^+ diffusion between ECs and bath solution $\big(\mu M s^{-1}\big)$ is

$$J_{KNEtoSC} = diff(K_{bath} - K_o), \quad (24)$$

where $diff$ is the diffusion constant.

The rate equations for the Na^+ concentration in the ECS depends on the flux through Na^+ channels,Na^+/K^+-ATPase, NKCC current, and Na^+ exchange with the bath solution.

$$\frac{dNa_o}{dt} = \frac{1}{VR_{sa}}\Big(J_{Na} + 3J_{NaK} - J_{NKCC} - \big(-3J_{NCX} + J_{NMDA_{Na}} + J_{AMPA_{Na}} + 3J_{GluT}\big) \times \rho_{conv}\Big) + J_{NaNEtoSC}. \quad (25)$$

Na^+ flux through Na^+ channels $(\mu M s^{-1})$:

$$J_{Na} = G_{Na}\left(v - E_{Na}\right). \quad (26)$$

G_{Na} is the whole-cell conductance of Na^+ channels and E_{Na} is the reversal potential for the Na^+.

$$E_{Na} = \frac{v_T}{z_{Na}} \ln\left(\frac{Na_o}{Na_a}\right). \quad (27)$$

The extracellular Na^+ also depends upon flux due to NCX, which is given by

$$J_{NCX} = I_{NCXmax} H_3\big(Na_o, K_{NCXmN}\big) H\big(Ca_o, K_{NCXmC}\big) \times \frac{\frac{Na_a^3}{Na_o^3} exp\big(\eta_{NCX} \times \frac{v_a}{v_T}\big) - \frac{Ca_a}{Ca_o} exp\big((\eta_{NCX} - 1) \times \frac{v_a}{v_T}\big)}{1 + k_{sat} exp\big((\eta_{NCX} - 1) \times \frac{v_a}{v_T}\big)}, \quad (28)$$

where I_{NCXmax} is the maximum current through NCX. K_{NCXmN}, and K_{NCXmC} are the binding affinities of Na^+ and Ca^{2+}, respectively, to NCX.

Na^+ flux through glutamate transporters is obtained by multiplying the K^+ flux due to transporters by a factor of 3 because the stoichiometry of EAAT is 3 Na^+: K^+:glu:H^+

Na^+ exchange with the bath solution $\big(\mu M s^{-1}\big)$ is

$$J_{NaNEtoSC} = diff \times (Na_{bath} - Na_o), \quad (29)$$

where diff is the diffusion constant of of Na^+.

Cl^- concentration in the ECS is (μM) is given by conservation of charge, that is

$$Cl_o = Na_o + K_o. \tag{30}$$

K^+ concentration in the astrocyte (K_a) depends on flux due to K^+ channel, Na^+/K^+ ATPase, NKCC, and BK channels.

$$\frac{dK_a}{dt} = -J_K + 2J_{NaK} + J_{NKCC} + J_{KCC} - J_{BK} - \big(J_{GluT} + J_{NMDA_K} + J_{AMPA_K}\big)\rho_{conv}. \tag{31}$$

With the exception of J_{BK}, all other fluxes are explained above. K^+ flux through the BK channels $(\mu M s^{-1})$ is

$$J_{BK} = G_{BK} w \left(v - E_{BK}\right), \tag{32}$$

where G_{BK} is the conductance of BK channels, and E_{BK} is the Nernst potential of BK channel (mV) given by

$$E_{BK} = \frac{v_T}{z} \ln\left(\frac{K_p}{K_a}\right), \tag{33}$$

z is the valence of K^+ and p represents the perivascular region.

Na_a depends on the flux through Na^+ channels, Na^+/K^+ ATPase, NKCC, NCX, NMDA receptors, AMPA receptors, and glutamate transporters. These fluxes are already explained in the above sections.

$$\frac{dNa_a}{dt} = -J_{Na} - 3J_N aK + J_{NKCC} - \big(3J_{NCX} - J_{NMDA_{Na}} - J_{AMPA_{Na}} - 3J_{GluT}\big) \times \rho_{conv}. \tag{34}$$

Cl^- concentration in the astrocyte is given by electroneutrality (μM).

$$\frac{dCl_a}{dt} = \frac{dNa_a}{dt} + \frac{dK_a}{dt} + 2\frac{dCa_a}{dt}, \tag{35}$$

Cytosolic Ca^{2+} (Ca_a) is regulated by IP_3 receptors-mediated release from the the endoplasmic reticulum (ER), release from the ER through leak channels, uptake by the ER through SERCA, influx from ECS through NMDA receptors and TRPV4 channels, and flux through NCX.

$$\frac{dCa_a}{dt} = B_{cyt}\Big(J_{IP3} - J_{pump} + J_{ERleak} - \frac{J_{TRPV}}{r_{buff}}\Big) + \big(J_{NCX} + J_{NMDA_{Ca}}\big) \times \rho_{conv}. \tag{36}$$

The Ca^{2+} buffering is described by steady-state approximation, i.e.

$$B_{cyt} = \left(1 + BK_{end} + \frac{K_{ex}B_{ex}}{\left(K_{ex} + Ca_a\right)^2}\right)^{-1}, \tag{37}$$

where BK_{end} is the ratio of endogenous buffer concentration to dissociation constant, K_{ex} is dissociation constant of exogenous buffer, and B_{ex} the concentration of an exogenous buffer.

The flux of Ca^{2+} through the IP_3R channels $(\mu M s^{-1})$ is

$$J_{IP3} = J_{max}\Big(H\big(IP_3, K_i\big)H\big(Ca_a, K_{act}\big)h\Big)^3\left(1 - \frac{Ca_a}{s}\right). \tag{38}$$

J_{max} is the maximum rate of Ca^{2+} through IP_3Rs, K_i dissociation constant for IP_3 binding to IP_3R, K_{act} dissociation constant for Ca^{2+} binding to an activation site on the IP_3R.

Ca^{2+} flux through SERCA pumps $(\mu M s^{-1})$ is

$$J_{pump} = V_{max} H_2 \big(Ca, K_{pump}\big). \tag{39}$$

V_{max} is the maximum rate of Ca^{2+} uptake by SERCA pumps and k_{pump} represents the Ca^{2+} dissociation constant of SERCA.

Ca^{2+} flux through the leak channels $(\mu M s^{-1})$ is

$$J_{ERleak} = P_L \left(1 - \frac{Ca_a}{s}\right). \tag{40}$$

P_L is the maximum flux through leak channels.

The flux through TRPV4 channels $(\mu M s^{-1})$ is

$$J_{TRPV} = G_{TRPV} m \left(v - E_{TRPV}\right), \tag{41}$$

where G_{TRPV} is the maximum conductance of the TRPV4 channels.

The Nernst potential for the current through TRPV4 channels (mV) is

$$E_{TRPV} = \frac{v_T}{z_{Ca}} \log \left(\frac{Ca_p}{Ca_a}\right). \tag{42}$$

z_{Ca} is the valency of Ca^{2+} and Ca_p is the Ca^{2+} in the perivascular space.

Ca^{2+} flux through NCX is 3 times smaller than the Na^+ given by $(eq.28)$ due to 3:1 stoichiometry. That is, NCX exchanges 3 Na^+ for 1 Ca^{2+}.

The astrocytic inositol 1,4,5-trisphosphate IP_3 concentration (μM) is given by the following rate equation.

$$\frac{dIP_3}{dt} = r_h G - k_{deg} IP_3. \tag{43}$$

r_h is the maximum rate of IP_3 production in astrocytes due to metabotropic glutamate receptors and K_{deg} is the rate constant for IP_3 degradation. The ratio of active to total G-proteins (G) is

$$G = \frac{\rho + \delta_G}{K_G + \rho + \delta_G}. \tag{44}$$

The ratio ρ of bound to unbound metabotropic receptors on the astrocytic process is

$$\rho = \rho_{min} + \frac{\rho_{max} - \rho_{min}}{glu_{max}} glu. \tag{45}$$

glu is the exogenously applied glutamate concentration. We apply 1 mM for 100 ms, which decays exponentially as discussed in the main text.

The astrocytic epoxyeicosatrienoic acid (EET) concentration (μM) is modeled by the following equation.

$$\frac{deet}{dt} = V_{eet} max\left(Ca_a - c_{k_{min}}, 0\right) - k_{eet} eet \tag{46}$$

V_{eet} and k_{eet} are the EET production and degradation rates, respectively.

Ca^{2+} concentration in the ER (μM) is

$$\frac{ds}{dt} = \frac{-B_{cyt}}{VR_{ER_{cyt}}} \left(J_{IP3} - J_{pump} + J_{ERleak}\right). \tag{47}$$

$VR_{ER_{cyt}}$ is the volume ratio between ER and cytosol.
The open probability of BK channels is

$$\frac{dw}{dt} = \phi_n \left(w_\infty - w\right), \tag{48}$$

where the time constant associated with the opening of BK channel is

$$\phi_n = \psi_n cosh\left(\frac{v - v_3}{2v_4}\right). \tag{49}$$

ψ_n is a characteristic time for the opening of BK channels.
The equilibrium state of BK channels is

$$w_\infty = \frac{1}{2}\left(1 + tanh\left(\frac{v + eet_{shift}eet - v_3}{v_4}\right)\right). \tag{50}$$

eet_{shift} describes the EET dependent voltage shift, and

$$v_3 = -\frac{v_5}{2} tanh\left(\frac{Ca_a - Ca_3}{Ca_4}\right) + v_6. \tag{51}$$

The inactivation variable h of IP_3R channels is

$$\frac{dh}{dt} = k_{on}\left[K_{inh} - \left(Ca_a + K_{inh}\right)h\right], \tag{52}$$

where k_{on} is the rate of Ca^{2+} binding to the inhibitory site of IP_3R and K_{inch} is dissociation constant of inhibitory site.
Membrane potential of the astrocyte (mV) is

$$\begin{aligned}\frac{dv_a}{dt} = \gamma_v \left(-J_{BK} - J_K - J_{Cl} - J_{Na} - J_{NaK} - 2J_{TRPV}\right) - \\ \left(-2J_{GluT} + J_{NCX} - J_{NMDA} - J_{AMPA}\right) \times \rho_{conv},\end{aligned} \tag{53}$$

where γ_v converts flux from concentration unit to current unit. The fluxes involve in the astrocytic membrane potential except J_{Cl} are already explained in the above section.
Cl^- flux through leak channels $(\mu M s^{-1})$ is

$$J_{Cl} = G_{Cl}\left(v - E_{Cl}\right). \tag{54}$$

G_{Cl} is the maximum conductance of Cl^- channels. E_{Cl} is the reversal potential of Cl^- and is given by

$$E_{Cl} = \frac{v_T}{z_{Cl}} \ln\left(\frac{Cl_o}{Cl_a}\right), \tag{55}$$

z_{cl} is the valence of Cl^-.

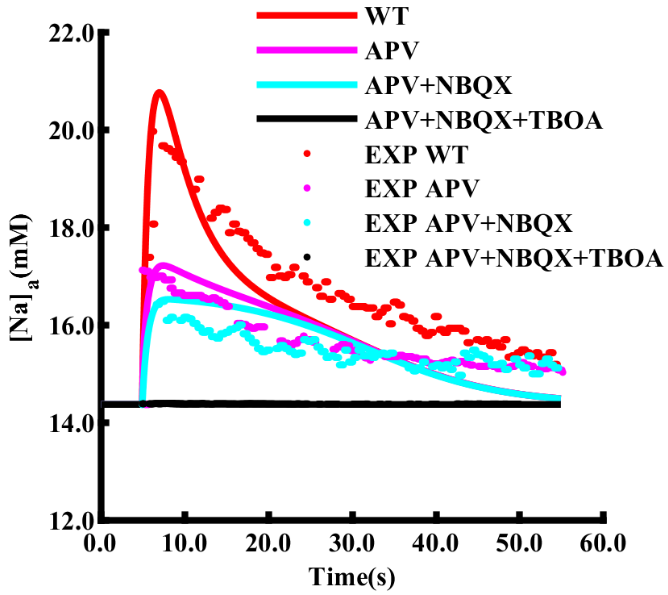

Fig S1: **Fits to Na_a time traces can be improved if different decay constants for the ligand in the cortex and hippocampus are used.** Same study as in Fig. 4Ai of the main text but different time scales for the glutamate decay are used in the cortex and hippocampus.

Table S1: Parameters for ion dynamics in the astrocyte and ECS and membrane potential of the astrocyte.

Parameter	Description	value
VR_{sa}	Volume ratio between the ECS and astrocyte	3
glu_{max}	Maximum glutamate concentration	1000 μM
G_K	Peak conductance of K^+ channels	6907.77 $\mu MmV^{-1}s^{-1}$
G_{Na}	Peak conductance of Na^+ channels	226.94 $\mu MmV^{-1}s^{-1}$
G_{NBC}	Peak conductance of the NBC cotransporter	130.74 $\mu MmV^{-1}s^{-1}$
G_{KCC}	Peak conductance of the KCC	1.728 $\mu MmV^{-1}s^{-1}$
G_{NKCC}	Peak conductance of the NKCC	9.568 $\mu MmV^{-1}s^{-1}$
G_{BK}	Peak conductance of BK channels	10.25 $\mu MmV^{-1}s^{-1}$
G_{Cl}	Peak conductance of Cl^{-1} leak	151.93 $\mu mV^{-1}s^{-1}$
$J_{NaK_{max}}$	Maximum flux through Na^+/K^+ ATPase in the cortex	2.84×10^4 μMs^{-1}
$J_{NaK_{max}}$	Maximum flux through Na^+/K^+ ATPase in the hippocampus	2.934×10^4 μMs^{-1}
K_{Na}	Association constant for Na^+ to Na^+/K^+ATPase	10×10^3 μM
K_{K_o}	Association constant for K^+ to Na^+/K^+ ATPase	1.5×10^3 μM
ρ_{min}	Minimum ratio of bound to unbound IP_3 receptors	0.1
ρ_{max}	Maximum ratio of bound to unbound IP_3 receptors	0.7
δ_G	Ratio of the activities of the unbound and bound receptors	1.235×10^{-2}
K_G	G-protein disassociation constant	8.82
r_h	Maximum rate of IP_3 production due to mGluRs	4.8 μMs^{-1}
K_{deg}	Rate constant in for IP_3 production	1.25 s^{-1}
r_{buff}	Rate of Ca^{2+} buffering at the endfeet compared to the astrocyte body	0.05
$VR_{ER_{cyt}}$	Volume ratio between the ER and astrocytic cytosol	0.185
BK_{end}	Ratio of endogenous buffer concentration to disassociation constant	40
K_{ex}	Disassociation constant of exogenous buffer	0.26 μM
B_{ex}	Concentration of exogenous buffer	11.35 μM
J_{max}	Maximum rate of Ca^{2+} through the IP_3Rs	2880 μMs^{-1}
K_i	Disassociation constant of IP_3 binding to IP_3R	0.03 μM
K_{act}	Disassociation constant of Ca^{2+} binding to the activation site of IP_3R	0.17 μM
k_{on}	Rate of Ca^{2+} binding to the inhibitory site of IP_3R	2 $\mu M^{-1}s^{-1}$
K_{inch}	Disassociation constant of IP_3R	0.1 μM
V_{max}	Maximum rate of SERCA	20 μMs^{-1}
k_{pump}	Disassociation constant of SERCA	0.24 μM
P_L	ER leak channel steady-state balance constant	0.0804 μMs^{-1}
V_{eet}	EET production rate	72 s^{-1}
c_{min}	Minimum Ca^{2+} concentration required for EET production	0.1 μM
k_{eet}	EET degradation rate	7.2 S^{-1}
v_4	Measure of the spread of W_∞	8 mV
eet_{shift}	Describe the EET dependent voltage shift	2 $mV\mu M^{-1}$
v_5	Determines the range of the shift of w_∞ as Ca^{2+} varies	15 mV
v_6	Shifts the range of w_∞	-55 mV
ψ_n	Characteristic time for the opening of the BK channel	2.664 s^{-1}
Ca_3	BK open probability Ca^{2+} constant	0.4
Ca_4	BK open probability Ca^{2+} constant	0.35 μM
Na_{bath}	Bath concentrations of Na^+	152 mM
K_{bath}	Bath concentration of K^+	2.6 mM
$diff$	Diffusion constant of Na^+ and K^+	0.5 s^{-1}

Table S2: Parameters for glutamate transporters and NCX.

Parameter	Description	value
$J_{GluT_{max}}$	Maximum glutamate transporters flux	$0.11\ pA\mu m^{-2}$
K_{GluTmN}	Rate of sodium binding to glutamate transporter	15000 μM
K_{GluTmK}	Rate of potassium binding to glutamate transporter	5000 μM
K_{GluTmg}	Rate of glutamate binding to glutamate transporter	34 μM
$J_{NCX_{max}}$	Maximum NCX current	$1\ pA\mu m^{-2}$
K_{NCXmN}	Half-saturation constant for sodium	875000 μM
K_{NCXmC}	Half-saturation constant for calcium	1380 μM
η_{NCX}	The position of energy barrier	0.35
$ksat$	Saturation factor	0.1

Table S3: The initial values of various variables.

Parameter	Description	value
Ca_o	Extracellular calcium concentration	2000 μM
Ca_a	Intracellular calcium concentration	0.12 μM
Na_o	Extracellular Na^+ concentration	149 mM
Na_a	Intracellular Na^+ concentration	14 mM
K_o	Extracellular K^+ concentration	2.9 mM
K_a	Intracellular K^+ concentration	96 mM
Cl_a	Intracellular Cl^- concentration	7.6 mM
v_a	Intracellular membrane potential	$-89\ mV$
IP_3	Initial IP_3 concentration	0.048 μM
EET_a	Initial EET concentration	0.70 μM
h_a	Initial value of the inactivation variable	0.55
s_a	Initial calcium concentration in astrocytic ER	466 μM
w_a	Initial value of open probability of BK channel	9×10^{-6}

Table S4: Parameters for NMDA and AMPA receptors' kinetics in the presence of glutamate.

Parameter	Description	value
k_{on}	Binding rate of agonist to NMDA receptor	0.01305 $mM^{-1}s^{-1}$
k_{off}	Unbinding rate of agonist to NMDA receptor	153.68531 s^{-1}
β	Opening rate of NMDA receptor	699.93257 s^{-1}
α	Closing rate of NMDA receptor	32.24297 s^{-1}
k_d	Desensitizing rate of NMDA receptor	93.39486 s^{-1}
k_r	Resensitizing rate of NMDA receptor	4.6334 $s^{-}1$
$J_{NMDA_{max}}$	Maximum NMDA current due to glutamate concentration	350 $pA\mu m^{-2}$
b_1	Voltage stabilizing factor 1 for current-voltage dependence	-0.033 mV^{-1}
b_2	Voltage stabilizing factor 2 for current-voltage dependence	0.007 mV^{-1}
R_b	Binding rate of agonist to AMPA receptor	0.098 $\mu M^{-1}s^{-1}$
R_{u1}	Unbinding rate of agonist to AMPA receptor for a single A-bound state	5.1016 s^{-1}
R_{u2}	Unbinding rate of agonist to AMPA receptor for double A-bound states	270.57 s^{-1}
R_d	Desensitizing rate of AMPA receptor	105.38 s^{-1}
R_r	Resensitizing rate of AMPA receptor	1.3353 s^{-1}
R_o	Opening rate of AMPA receptor	29000 s^{-1}
R_c	Closing rate of AMPA receptor	146 s^{-1}
$convg$	Converts $\frac{pA}{\mu m^2}$ to $\frac{\mu M}{s}$	13605.076 $\frac{\mu M}{s^{-1}}\frac{pA^{-1}}{\mu m^2}$
$J_{AMPA_{max}}$	Maximum AMPA current due to glutamate activation	350 $pA\mu m^{-2}$

Table S5: Parameters for NMDA receptor's kinetics in the presence of NMDA.

Parameter	Description	value
k_{on}	Binding rate of agonist to NMDA receptor	0.0043305 $mM^{-1}s^{-1}$
k_{off}	Unbinding rate of agonist to NMDA receptor	49.81 s^{-1}
β	Opening rate of NMDA receptor	500.10 s^{-1}
α	Closing rate of NMDA receptor	33.903 s^{-1}
k_d	Desensitizing rate of NMDA receptor	88.75 s^{-1}
k_r	Resensitizing rate of NMDA receptor	4.81 $s^{-}1$
$J_{NMDA_{max}}$	Maximum NMDA current due to NMDA concentration	350 $pA^{-}1\mu m^2$
b_1	Voltage stabilizing factor 1 for current-voltage dependence	-0.038 mV^{-1}
b_2	Voltage stabilizing factor 2 for current-voltage dependence	0.011 mV^{-1}
